# Comparative Transcriptomic Analysis of Virulence Factors in *Leptosphaeria maculans* during Compatible and Incompatible Interactions with Canola

**DOI:** 10.3389/fpls.2016.01784

**Published:** 2016-12-01

**Authors:** Humira Sonah, Xuehua Zhang, Rupesh K. Deshmukh, M. Hossein Borhan, W. G. Dilantha Fernando, Richard R. Bélanger

**Affiliations:** ^1^Département de Phytologie, Faculté des Sciences de l'Agriculture et de l'Alimentation, Université LavalQuébec QC, Canada; ^2^Department of Plant Science, University of Manitoba WinnipegWinnipeg, MB, Canada; ^3^Agriculture and Agri-Food CanadaSaskatoon, SK, Canada

**Keywords:** *Avr* genes, CAZymes, compatible interactions, effectors, incompatible interactions, RNA-seq transcriptome profiling

## Abstract

*Leptosphaeria maculans* is a hemibiotrophic fungus that causes blackleg of canola (*Brassica napus*), one of the most devastating diseases of this crop. In the present study, transcriptome profiling of *L. maculans* was performed in an effort to understand and define the pathogenicity genes that govern both the biotrophic and the necrotrophic phase of the fungus, as well as those that separate a compatible from an incompatible interaction. For this purpose, comparative RNA-seq analyses were performed on *L. maculans* isolate D5 at four different time points following inoculation on susceptible cultivar Topas-DH16516 or resistant introgression line Topas-*Rlm2*. Analysis of 1.6 billion Illumina reads readily identified differentially expressed genes that were over represented by candidate secretory effector proteins, CAZymes, and other pathogenicity genes. Comparisons between the compatible and incompatible interactions led to the identification of 28 effector proteins whose chronology and level of expression suggested a role in the establishment and maintenance of biotrophy with the plant. These included all known *Avr* genes of isolate D5 along with eight newly characterized effectors. In addition, another 15 effector proteins were found to be exclusively expressed during the necrotrophic phase of the fungus, which supports the concept that *L. maculans* has a separate and distinct arsenal contributing to each phase. As for CAZymes, they were often highly expressed at 3 dpi but with no difference in expression between the compatible and incompatible interactions, indicating that other factors were necessary to determine the outcome of the interaction. However, their significantly higher expression at 11 dpi in the compatible interaction confirmed that they contributed to the necrotrophic phase of the fungus. A notable exception was *LysM* genes whose high expression was singularly observed on the susceptible host at 7 dpi. In the case of TFs, their higher expression at 7 and 11 dpi on susceptible Topas support an important role in regulating the genes involved in the different pathogenic phases of *L. maculans*. In conclusion, comparison of the transcriptome of *L. maculans* during compatible and incompatible interactions has led to the identification of key pathogenicity genes that regulate not only the fate of the interaction but also lifestyle transitions of the fungus.

## Introduction

Blackleg disease (stem canker) caused by *Leptosphaeria maculans* (Desm.) Ces. & De Not. is one of the major constraints to canola (*Brassica napus* L.) production worldwide (Fitt et al., 2006). Infection by the fungus is known to cause more than 50% yield losses in canola (Kutcher et al., 2013). The major difficulty for combating the pathogen lies in the understanding of its complex lifestyle, which includes alternative biotrophic, and necrotrophic, phases, along with a symptomless endophytic phase (Howlett et al., 2001; Van de Wouw et al., 2016). Management of blackleg disease includes crop rotations, seed treatment and fungicide applications, and preferably, disease-resistant cultivars, arguably the most effective approach (Delourme et al., 2006).

Canola shows two types of resistance against *L. maculans*: qualitative and quantitative. Single or few major genes that are known to be involved in a gene for gene interaction govern qualitative resistance. Major genes provide resistance particularly at the seedling stage whereas quantitative resistance involves many small effect genes that are mostly expressed during the adult plant stage (Raman et al., 2012; Huang et al., 2014). To date, several resistance genes with major effect have been identified in *Brassica* species, but only two, *LepR3* and *Rlm2*, have been cloned and well characterized (Delourme et al., 2006; Long et al., 2011; Larkan et al., 2013, 2015; Van de Wouw et al., 2014). On the other hand, better progress has been achieved with *L. maculans* where 14 avirulence genes have been identified, and seven of them, namely *AvrLm1, AvrLm4-7, AvrLm6, AvrLm11, AvrLmJ1, AvrLm2*, and *AvrLm3* have been cloned (Gout et al., 2006; Fudal et al., 2007; Parlange et al., 2009; Balesdent et al., 2013; Van de Wouw et al., 2014, 2016; Ghanbarnia et al., 2015; Plissonneau et al., 2016). Interestingly, some of these avirulence genes have been found to be clustered, with clusters *AvrLm1*-2-6 and *AvrLm3*-4-7-9-*AvrLepR1* being the notable examples (Balesdent et al., 2002; Ghanbarnia et al., 2012). For the most part, avirulence genes, including *L. maculans Avrs*, are small-secreted proteins (SSPs) with several cysteine residues, and often referred to as effectors (Stukenbrock and McDonald, 2009; Rouxel et al., 2011).

Effectors are key elements in fungal virulence against plants and particularly important during the biotrophic phase of infection (Kloppholz et al., 2011). *L. maculans*, being a hemibiotroph, will initially rely on effectors to suppress plant defenses, and then will subsequently use effectors to kill plant cells. In *L. maculans*, most putative or candidate effectors are localized in transposon-rich repetitive DNA and are affected by a repeat-induced point mutation (Rouxel et al., 2011). The putative effector genes are mostly over-expressed during primary leaf infection (Soyer et al., 2014). Such information about the genomic localization, gene organization, and expression dynamics is helpful to understand the host-pathogen interaction and more particularly for the identification of *bona fide* effectors. Similarly, transcription factors (TFs), and carbohydrate active enzymes (CAZymes) are known to play a pivotal role in host-pathogen interactions, and are, along with effectors, prime targets for studying virulence factors in fungi (Guo et al., 2011; Lombard et al., 2014; Lowe et al., 2014; Malinovsky et al., 2014).

Transcription factors are essential players in the signal transduction pathways. In *L. maculans*, TF LmStuA is found to be required for normal growth, perithecium formation, pathogenicity on oilseed rape leaves, and expression of effectors (Soyer et al., 2015). The silencing of *LmStuA* triggers drastic effects on the morphogenesis and pathogenicity of *L. maculans*, indicating that it may affect a large number of genes and pathways (Soyer et al., 2015). Similarly, several CAZymes in *L. maculans* genome have been predicted to have a functional role in pathogenesis (Lowe et al., 2014). CAZymes are important to break down the polysaccharides of plant cell walls, to establish infection, and also, to facilitate access to nutrients during the necrotrophic and saprophytic growth phases. For instance, global transcriptomic analyses of the hemibiotroph *Colletotrichum higginsianum* revealed that genes encoding secreted proteins without a functional annotation are expressed predominantly during the initial biotrophic phase, whereas expression of secreted lytic enzymes (including CAZymes) was higher in the subsequent necrotrophic phase (O'Connell et al., 2012). A similar finding was observed in *Leptosphaeria biglobosa*, a necrotroph expressing more cell wall degrading genes than *L. maculans* (Lowe et al., 2014). However, *L. maculans* expressed many genes in the carbohydrate binding module (CBM) class of CAZymes, particularly CBM50 genes, during early infection, and cell wall degrading enzymes at later stages of growth (Lowe et al., 2014). This suggests that expression of secreted proteins without functional annotation is a general feature of biotrophy, whereas expression of cell wall degrading enzymes is generally associated with necrotrophy. Other important necrotrophy-related genes code for sirodesmin PL (Sir), a phytotoxin that belongs to the class of epipolythiodioxopiperazine (ETP). The production of sirodesmin by *L. maculans* is thought to be suppressed by brassinin, a phytoalexin of canola (Pedras et al., 1993). In *L. maculans*, a cluster of 23 genes including polyketide synthase (PKS), non-ribosomal peptide synthase (NRPs) genes, and 18 Sir genes have been identified (Gardiner et al., 2004).

The infection process is highly dependent on host recognition and molecular cross-talk between the host and the pathogen where pathogenicity-related genes play an important role. However, any given host-pathogen interaction is a very complex phenomenon, which makes it difficult to understand the factors dictating compatibility or incompatibility. In Arabidopsis, Huibers et al. (2009) were able to discriminate genes induced during compatible from incompatible interactions with the downy mildew pathogen *Hyaloperonospora arabidopsidis* (downy mildew). Similarly, other studies have been conducted to compare gene expression profiling under compatible and incompatible interactions (Wang et al., 2010; Sestili et al., 2011; Li et al., 2015).

Expression profiling during the development of disease is an effective approach to better understand the pathogenesis process. Current improvements in sequencing technologies have provided new opportunities to evaluate gene expression under different conditions by sequencing the entire transcriptome (Wang et al., 2009). Compared to other hybridization based transcriptome profiling platforms like microarrays, RNA-seq provides access to simultaneous transcript discovery and abundance estimation, identification of differentially-expressed genes (DEGs) and associated molecular cellular pathways, and alternative splicing variants (Wang et al., 2009; Trapnell et al., 2012). However, RNA-seq analysis requires scalable, fast, and statistically relevant software tools that can handle complex and large sequence data. Fortunately, considerable efforts have been devoted to the development of specialized software tools to perform effective RNA-seq analysis (Garber et al., 2011; Trapnell et al., 2012; Seyednasrollah et al., 2015; Sonah et al., 2016).

The *L. maculans*-canola compatible interaction has been previously addressed using the RNA-seq approach (Lowe et al., 2014; Haddadi et al., 2016). These studies made significant efforts toward the understanding of susceptible reaction in canola after *L. maculans* infection and provides the first in-depth look into the transcriptomic profile of this interaction. In this study, our objective was to compare the pathogen responses against susceptible and resistant host genotypes in order to obtain a precise definition of the virulence factors expressed by *L. maculans* during its biotrophic and necrotrophic phase. For this purpose, we performed the RNA-seq transcriptome profiling of *L. maculans* inoculated on susceptible and resistant canola lines at four developmental stages over five biological replications. A particular emphasis was placed on the identification of DEGs including effectors, CAZymes, and other pathogenesis-related genes during blackleg disease development in canola.

## Materials and methods

### Plant material and *L. maculans* inoculation

Canola (*B. napus*) breeding lines, Topas DH16516 (Topas-wild), a double haploid line susceptible to *L. maculans* and Topas*-Rlm2*, an introgression line resistant to *L. maculans* isolates carrying *AvrLm2* (Larkan et al., 2016a,b) were used as plant material. Seven-day old seedlings of both resistant and susceptible canola lines grown under controlled environment were point inoculated with pycnidiospores suspension of *L. maculans* isolate D5. *L. maculans* isolate D5 contains known avirulence effectors *AvrLm1, AvrLm4-7, AvrLm2*, and *AvrLmJ1* but lacks *AvrLm6* (Raman et al., 2012). Five biological replicates each with leaves from eight plants per sample were collected at 3, 5, 7, and 11 days post inoculation (dpi). The *L. maculans* isolate D5 was cultured in V8® agar plates at 25°C. For RNA extraction, two fungal agar plugs were inoculated in V8® liquid media and incubated at 22°C. After 7 days, the mycelium was harvested for RNA extraction.

### Sample preparation and illumina sequencing

Total RNA for all samples was extracted using a combined Trizol/Qiagen RNeasy mini kit (Qiagen, Mississauga, ON, Canada). The RNA quality and quantity was accessed using NanoDrop ND-1000 spectrophotometer (NanoDrop technologies) and further verified by agarose gel electrophoresis. Four micrograms of total RNA from all samples were used to make individual barcoded cDNA library using TruSeq RNA library preparation kit v2 with some modifications. Individual library was assessed using the Agilent 2100 Bioanalyzer (Agilent Technologies). The products resulted in a smear with an average fragment size of approximately 260 bp. A total of six individual libraries were pooled using the uniform amount of each library and the quality of the final library pools was also assessed. Single end, 100 bp sequencing was performed using an Illumina HiSeq 2000 sequencer at the Génome Québec Innovation Centre (McGill University, Quebec, Canada).

### RNA-seq data analysis

RNA-seq reads were quality-checked with fastqc, which performs various quality checks for the raw reads. Read processing was performed by using Trimmomatic software (Bolger et al., 2014). Reads were trimmed from both ends until the average of all 5 bp sliding windows reached a Phred score of 25 or higher and all the sequences shorter than 35 bases were discarded. Processed reads were aligned to the *L. maculans* genome and transcriptome with Tophat2 (Trapnell et al., 2009). Most of the parameters in Tophat was set as default. A mismatch of two bases were allowed for the alignment. The minimum and maximum intron length was set to 50 and 500,000 respectively. Reads aligned to multiple sites were removed prior to further analysis.

Novel transcripts that did not overlap with any annotated transcripts were identified using Cufflink tools. The gene expression level for each annotated as well as non-annotated novel transcripts were estimated as the number of Fragments (reads) per kilobase of transcript per million mapped reads (FPKM), considering only uniquely mapped reads in exonic region by using the Cufflink software. The differentially expressed genes (DEGs) were identified using four different tools including Cuffdiff (Trapnell et al., 2012), EdgeR (Robinson et al., 2010), DESeq2 (Love et al., 2014), and CLC Genomics Workbench. We used FDR < 0.0001 and the absolute value of log_2_ (Fold-change) >1.5 relative to axenic culture as the threshold for the identification of DEGs. We used quartile normalization that excludes the top 25% of expressed genes to improve detection of less abundant genes. With the Cufflink software, we used -M option to mask rRNA, -b, and -u option for bias correction and option to normalize for aligned tags instead of total tags. HTseq tool was used to count reads prior to DEGs identification. The DESeq2 package was used to estimate sample quality (PCA) and the expression level of transcripts. The regularized rlog transformation and variance stabilizing transformation were used for data visualization. Time course analysis for all the four time points were carried out to find the genes that reacted in a time-specific manner using DESeq2 package.

### Functional annotation and gene ontology

Standard gene ontology (GO) was used to describe DE gene functionality, a hypergeometric test and the *p* < 0.05 of Pearson Chi-Square test between the gene numbers of the two input dataset were used to map the DT genes to GO terms based on the BGI WEGO (Web Gene Ontology Annotation Plot, http://wego.genomics.org.cn/cgi-bin/wego/index.pl). Single enrichment analysis (SEA), a function of AgriGO was used to examine GO term enrichment using *Magnaporthe grisea* as a background reference using Fisher statistical test and 0.05 significance level and other default parameters. Transcription factors were identified using Fungal Transcription Factor Database (FTFD) (Park et al., 2008). The FTFD pipeline sorts fungal TFs initially based on the relevant InterPro terms like DNA-binding motifs, and then the false-positive TFs are filtered with different criteria. CAZymes were identified using dbCAN server (Yin et al., 2012). The dbCAN hosted an analytical pipeline compiled with CDD (conserved domain database) search, family specific hidden Markov model and literature curation (Yin et al., 2012). For the classification of putative secreted peptidases, the sequences for the secreted proteins predicted by WoLF PSORT (cutoff score = 15) were submitted to MEROPS Batch Blast analysis (http://merops.sanger.ac.uk, Rawlings et al., 2014).

### Identification of candidate secreted effector proteins (CSEPs)

Predicted protein sequences from the *L. maculans* genome were retrieved from the JGI MycoCosm (http://genome.jgi.doe.gov/Lepmu1/Lepmu1.home.html) (Rouxel et al., 2011; Grigoriev et al., 2014). SignalP (cutoff probability = 0.8), TargetP (cutoff probability = 0.8), Psort (cutoff score = 15), BlastP (cutoff *e* = 1e^−05^), and TMHMM software tools along with Secretool pipeline were used to predict small-secretory proteins (Petersen et al., 2011; Cortázar et al., 2014). To prioritize candidate effector genes, the entire secretome was analyzed by EffectorP a machine learning method optimized for the prediction of fungal effectors (Sperschneider et al., 2015). Crinkler type effectors were also searched in the *L. maculans* genome with the FEMO software tool implemented in MEME suit by using conserved domain LFLAK (http://meme-suite.org/tools/fimo). Proteins with LFLAK-domain within the initial 100 AA were considered for further analysis. To verify the search parameters, Crinkler effector search was also performed in *Phytophthora sojae* genome and compared with earlier report by Haas et al. (2009). After an initial search with FIMO, candidate Crinkler effectors were analyzed with secretool pipeline and subsequently with effectorP software tool.

## Results

### Disease progress on compatible and incompatible host

**Figure 1:**
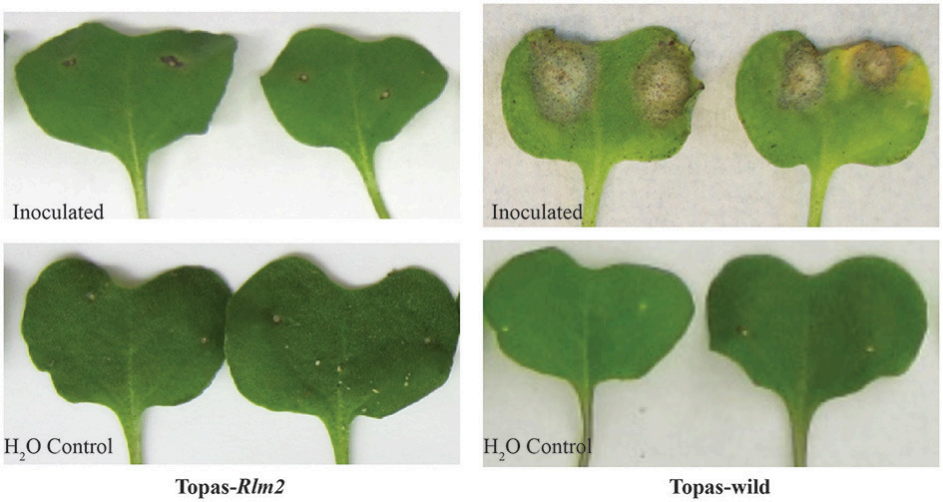
**Disease symptoms at 11 days post inoculation on leaves of compatible (Topas-wild) and incompatible (Topas*-Rlm2*) canola host inoculated with *Leptosphaeria maculans* D5 isolate or water**.

On plants of either the compatible host Topas-wild or the introgression line Topas*-Rlm2* carrying a major resistance gene, disease symptoms were not observed until 7 dpi. After this asymptomatic early growth stage, symptoms became visible exclusively on Topas-wild plants expanding into clear lesions and zones of chlorotic tissues at 11 dpi. In the case of Topas*-Rlm2* plants, there was no visible lesion beyond the site of inoculation (Figure 1). The disease progress on susceptible Topas-wild cotyledons caused by *L. maculans* was similar to that previously described on Westar (Lowe et al., 2014).

### Transcriptome sequencing with the time course of disease progress

**Figure 2:**
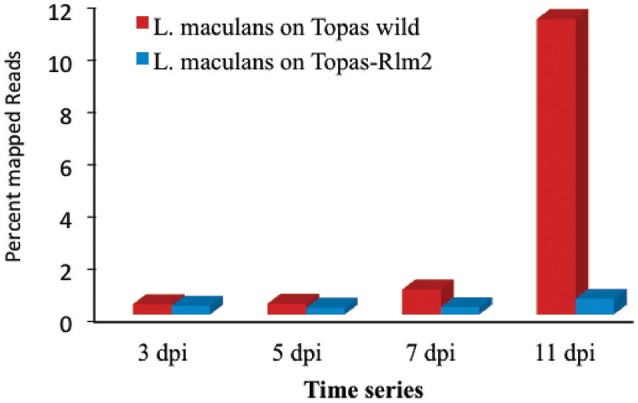
**Percentage of mapped reads to the *Leptosphaeria maculans* genome at 3, 5, 7, and 11 days post inoculation (dpi)**. RNA-seq was performed for *L. maculans* isolate D5 inoculated to Topas-wild (compatible) and Topas*-Rlm2* (incompatible) canola genotypes.

A total of 1.6 billion single-end reads consisting of approximately an average of 33 million reads for each cDNA library were obtained. The entire data set was submitted to SRA NCBI database and can be accessed with accession number SRP078092. The raw reads obtained were uniform across the different libraries. After performing quality assessment and read processing, about 0.1% of the reads with poor quality and shorter length were discarded. Mapping of the processed reads to the *L. maculans* genome showed a very high percentage of mapping for the axenic samples (Supplementary Table 1). The percentage of reads from *in planta* samples mapped to the *L. maculans* reference genome increased over time from <0.5% at 3 dpi to about 12% at 11 dpi in compatible host Topas-wild (Figure 2, Supplementary Table 1). By contrast, mapped fungal reads remain stable in the incompatible interaction with Topas*-Rlm2* and were well below 1% even at 11 dpi (Figure 2, Supplementary Table 1), a result well in line with the phenotype observed in Figure 1. Comparing the percent of mapped reads in the early asymptomatic growth stage in Topas-wild plants, only a marginal increase was observed between 3 and 5 dpi. This percentage more than doubled between 5 and 7 dpi and registered a 10-fold increase from 7 to 11 dpi (Supplementary Table 1).

**Figure 3:**
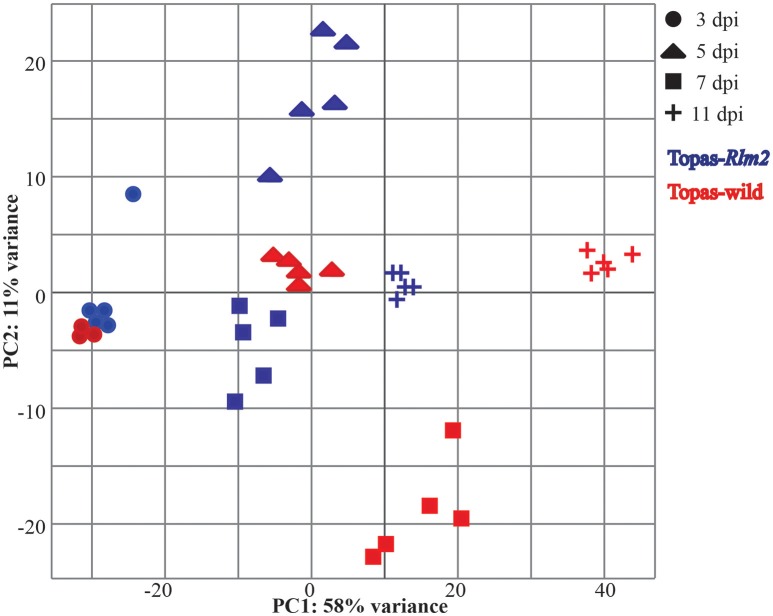
**Principal components (PCs) of RNA-seq expression profile obtained for *Leptosphaeria maculans in planta* samples during compatible and incompatible interactions**. A total of five biological replications for 3, 5, 7, and 11 days post inoculation (dpi) were used for RNA-seq expression profiling of *L. maculans* inoculated to compatible host Topas-wild (blue) and incompatible host Topas*-Rlm2* (Red).

The principal components (PCs) analysis highlighted a clear differential effect of the treatments along with the uniformity of the five biological replications within a treatment (Figure 3). The first principal component explained 58% of the expression variation supporting the large phenotypic differences between the conditions over the time period.

### Comparison of gene expression profiling during *in vitro* and *in planta* growth of *L. maculans*

**Figure 4:**
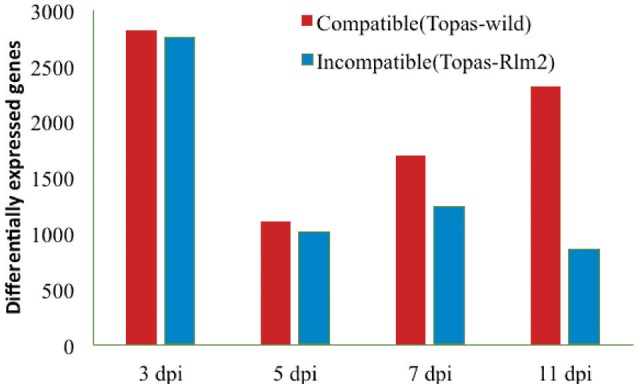
**Differentially expressed *Leptosphaeria maculans* genes identified at 3, 5, 7, and 11 dpi during compatible and incompatible interactions**. Differentially expressed genes were identified by comparing *in planta* samples with axenic cultures. Analyses were performed with five biological replicates and a threshold value of FDR < 0.0001 and log_2_ fold-change > 1.5.

The number of DEGs in *L. maculans* between *in vitro* axenic samples and samples at different *in planta* growth stages showed an interesting pattern of gene expression turnover over time. The overall pattern of DEGs identified with different software tools was similar (Supplementary Figure 1). The highest number of DEGs for both the compatible (Topas-wild) and incompatible (Topas*-Rlm2*) interactions was recorded in the early events (3 dpi) and was similar in both cases (Figure 4). This number was reduced by around three-fold at 5 dpi in both interactions and remained fairly level over the next sampling times in Topas*-Rlm2* plants. On the other hand, it increased steadily in Topas-wild plants to exceed by roughly three times the number of DEGs found in Topas*-Rlm2* plants (Figure 4).

**Figure 5:**
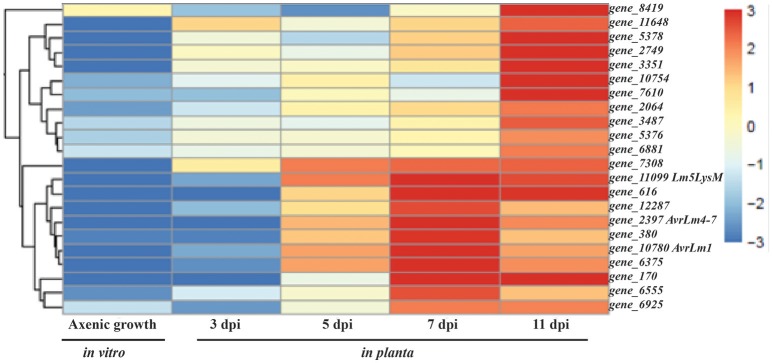
**Heat map of time series analysis showing expression pattern of the top 22 genes in *Leptosphaeria maculans* during compatible interaction with host Topas-wild**. Gene_2397, gene_10780, and gene_11099 represents *AvrLm4*-7, *AvrLm1*, and *Lm5LysM*, respectively. Analyses were performed with five biological replicates.

When looking at the top 20 upregulated DEGs at 3 dpi in the compatible interaction (Topas-wild), most of them were linked to uncharacterized proteins for which functional annotation was not available (Table 1). Since the uncharacterized proteins are unique to *L. maculans*, homology-based annotation failed to characterize them. Genes involved in chitin binding, fasciclin, and related adhesion glycoproteins known to play roles in early stages of infection were found to be highly upregulated (Table 1). Interestingly, of the top 20 upregulated genes of *L. maculans* identified during the compatible interaction with Topas-wild, 18 were also found associated with the incompatible interaction (Topas*-Rlm2*) (Table 1, Supplementary Table 2). However, the number of common genes gradually decreased over time to 11, 6, and 4 at 5, 7, and 11 dpi, respectively (Table 1, Supplementary Table 2). Time series analysis showed high expression of *Avr* genes during the biotrophic phase at 7 dpi while most of the highly expressed genes during the necrotrophic phase at 11 dpi were associated with molecular functions involved in catalase activity, hydrolases, CAZymes, peptidases, and transporters (Table 1, Figure 5).

**Table 1:** **List of the top 20 upregulated *Leptosphaeria maculans* genes during the compatible interaction with canola cultivar Topas-wild at each of the four sampling points**.

**Gene name**	**Gene ID**	**Locus**	**FPKM**	**SE**	^*^Log_2_ (FC)	**Padj**	**Functional annotations**
**3 dpi**							
Gene_11912	Lema_T061640	lm_SuperContig_8_v2:1610355–1610701	12276	1400.46	13.04	0.00E+00	Hypothetical protein
Gene_4884	Lema_T109090	lm_SuperContig_18_v2:862571–862990	14727	2585.81	10.89	0.00E+00	Hypothetical protein
Gene_9702	Lema_T050310	lm_SuperContig_5_v2:114784–120578	1424	229.89	10.79	0.00E+00	Chitin binding/Carbohydrate-Binding Module Family 18
Gene_1799	Lema_T087550	lm_SuperContig_11_v2:702412–703074	8854	1176.77	10.36	0.00E+00	Hypothetical protein (CSEP)
Gene_8310	Lema_T032040	lm_SuperContig_2_v2:2602620–2603501	1040	261.13	10.24	7.00E-159	Hypothetical protein
Gene_11624	Lema_T058760	lm_SuperContig_8_v2:828284–829346	1890	257.48	10	0.00E+00	Hypothetical protein SSP(Effector)
Gene_6862	Lema_T118070	lm_SuperContig_21_v2:752639–754522	1097	214.03	9.74	0.00E+00	G-protein coupled receptor activity
Gene_2936	Lema_T094030	lm_SuperContig_14_v2:218210–219154	1330	153.58	9.56	0.00E+00	Hypothetical protein
Gene_9395	Lema_T042140	lm_SuperContig_4_v2:1136979–1138783	1911	277.28	9.48	0.00E+00	Alcohol dehydrogenase
Gene_6482	Lema_T114270	lm_SuperContig_20_v2:529112–533279 (-)	6078	667.23	8.52	0.00E+00	Hypothetical protein
Gene_8982	Lema_T038010	lm_SuperContig_3_v2:2203740–2205537	1054	150.79	8	0.00E+00	Transporter activity
Gene_11976	Lema_T062280	lm_SuperContig_8_v2:1760041–1760879	2812	828.34	7.86	0.00E+00	Fasciclin and related adhesion glycoproteins
Gene_7649	Lema_T025430	lm_SuperContig_2_v2:804099–805458	1215	335.02	7.7	0.00E+00	Predicted transporter
Gene_11804	Lema_T060560	lm_SuperContig_8_v2:1317923–1319474	1108	232.60	7.65		Hypothetical protein SSP(Effector)
Gene_5070	Lema_T110950	lm_SuperContig_19_v2:299282–300301	1331	384.38	7.19	9.90E-111	Hypothetical protein
Gene_3811	Lema_T093240	lm_SuperContig_15_v2:1293850–1295649	1757	427.33	7.14	0.00E+00	Transporter activity
Gene_5572	Lema_T015460	lm_SuperContig_1_v2:975571–976381	1529	191.58	6.44	0.00E+00	Hypothetical protein
Gene_6173	Lema_T021470	lm_SuperContig_1_v2:2566440–2566907	1504	256.68	6.41	5.90E-221	Hypothetical protein
Gene_2652	Lema_T079530	lm_SuperContig_13_v2:633177–634702	2616	398.51	6.26	0.00E+00	Hypothetical protein
Gene_1331	Lema_T073560	lm_SuperContig_10_v2:445682–445936	1122	371.94	6.22	0.00E+00	Hypothetical protein
**5 dpi**							
Gene_4884	Lema_T109090	lm_SuperContig_18_v2:862571–862990	33992	1715.82	13.06	0.00E+00	Hypothetical protein
Gene_10161	Lema_T054900	lm_SuperContig_5_v2:1424583–1425111	1399	427.40	12.98	2.50E-90	Hypothetical protein
Gene_2216	Lema_T084480	lm_SuperContig_12_v2:827863–828642	1931	368.22	11.52	3.90E-96	Predicted protein SSP(Effector)
Gene_10273	Lema_T056020	lm_SuperContig_5_v2:1712896–1714128	1282	381.49	11.44	0.00E+00	Transcription factor
Gene_8310	Lema_T032040	lm_SuperContig_2_v2:2602620–2603501	1143	435.53	11.34	2.20E-214	Oxidoreductase activity
Gene_9395	Lema_T042140	lm_SuperContig_4_v2:1136979–1138783	1072	263.45	9.61	0.00E+00	Alcohol dehydrogenase
Gene_11804	Lema_T060560	lm_SuperContig_8_v2:1317923–1319474	2104	547.85	9.55	0.00E+00	Hypothetical protein (CSEP)
Gene_6173	Lema_T021470	lm_SuperContig_1_v2:2566440–2566907	4527	730.72	8.97	0.00E+00	Hypothetical protein
Gene_6375	Lema_T113200	lm_SuperContig_20_v2:228262–228840	1117	482.45	8.47	1.60E-196	Hypothetical protein (CSEP)
Gene_1799	Lema_T087550	lm_SuperContig_11_v2:702412–703074	1209	279.42	8.46	1.70E-289	Hypothetical protein (CSEP)
Gene_11976	Lema_T062280	lm_SuperContig_8_v2:1760041–1760879	1441	225.72	7.87	0.00E+00	Fasciclin and related adhesion glycoproteins
Gene_6483	Lema_T114280	lm_SuperContig_20_v2:534387–534664	1243	384.15	7.2	0.00E+00	Hypothetical protein
Gene_8491	Lema_T033100	lm_SuperContig_3_v2:491445–491728	1283	356.20	6.66	3.50E-20	Hypothetical protein
Gene_1613	Lema_T076380	lm_SuperContig_10_v2:1509441–1509941	2525	1043.94	6.4	7.30E-75	Hypothetical protein (CSEP)
Gene_616	Lema_T006160	lm_SuperContig_0_v2:2286680–2287371	1062	295.59	6.35	5.50E-208	Hypothetical protein
Gene_7940	Lema_T028340	lm_SuperContig_2_v2:1614035–1614898	4379	1327.86	6.33	0.00E+00	Transporter activity
Gene_415	Lema_T004150	lm_SuperContig_0_v2:1345345–1345518	1090	232.24	6.14	0.00E+00	Aminotransferase/Catalytic activity
Gene_6466	Lema_T114110	lm_SuperContig_20_v2:490947–492054	1030	361.07	5.94	1.90E-196	Ion channel activity
Gene_2652	Lema_T079530	lm_SuperContig_13_v2:633177–634702	1060	337.82	5.92	4.20E-292	Hypothetical protein
Gene_234	Lema_T002340	lm_SuperContig_0_v2:882882–883215	1670	221.35	5.58	1.00E-18	Hypothetical protein
**7 dpi**							
Gene_6534	Lema_T114790	lm_SuperContig_20_v2:655034–655196	18463	3470.09	18.02	1.30E-126	Predicted protein
Gene_10161	Lema_T054900	lm_SuperContig_5_v2:1424583–1425111	2763	185.07	13.08	1.30E-130	Hypothetical protein
Gene_2216	Lema_T084480	lm_SuperContig_12_v2:827863–828642	5389	739.19	12.12	3.80E-162	Predicted protein SSP(Effector)
Gene_1515	Lema_T075400	lm_SuperContig_10_v2:1026907–1027349	1157	262.25	11.46	2.50E-193	Hypothetical protein
Gene_8929	Lema_T037480	lm_SuperContig_3_v2:1850027–1850254	3062	510.96	11.38	6.90E-256	Hypothetical protein
Gene_11912	Lema_T061640	lm_SuperContig_8_v2:1610355–1610701	2410	453.43	10.77	1.20E-221	Hypothetical protein
Gene_2066	Lema_T082980	lm_SuperContig_12_v2:398032–399219	2890	507.44	10.73	7.10E-274	Hypothetical protein
Gene_8487	Lema_T033060	lm_SuperContig_3_v2:287922–288676	5468	1962.05	10.62	0.00E+00	Hypothetical protein (CSEP)
Gene_10780	Lema_T049660	lm_SuperContig_6_v2:1607018–1607681	3553	316.20	9.31	0.00E+00	Hypothetical protein (AvrLm1)
Gene_170	Lema_T001700	lm_SuperContig_0_v2:571327–571980	1695	466.93	9.31	0.00E+00	Hypothetical protein (CSEP)
Gene_6375	Lema_T113200	lm_SuperContig_20_v2:228262–228840	3556	1067.76	9.26	0.00E+00	Hypothetical protein (CSEP)
Gene_9004	Lema_T038230	lm_SuperContig_3_v2:2336899–2337410	23963	3704.18	9.06	0.00E+00	Hypothetical protein (CSEP)
Gene_616	Lema_T006160	lm_SuperContig_0_v2:2286680–2287371	9230	1311.26	8.58	0.00E+00	Hypothetical protein
Gene_4884	Lema_T109090	lm_SuperContig_18_v2:862571–862990	1940	697.48	8.04	4.40E-135	Hypothetical protein
Gene_2397	Lema_T086290	lm_SuperContig_12_v2:1374587–1375064	2131	209.12	8.04	0.00E+00	Hypothetical protein (AvrLm4-7)
Gene_1613	Lema_T076380	lm_SuperContig_10_v2:1509441–1509941	11945	2426.45	7.76	0.00E+00	Hypothetical protein (CSEP)
Gene_7362	Lema_T123070	lm_SuperContig_25_v2:296506–296973	1952	519.68	7.66	5.40E-226	Hypothetical protein (CSEP)
Gene_234	Lema_T002340	lm_SuperContig_0_v2:882882–883215	12153	4475.87	7.56	0.00E+00	Hypothetical protein
Gene_1799	Lema_T087550	lm_SuperContig_11_v2:702412–703074	1064	240.87	7.38	0.00E+00	Hypothetical protein (CSEP)
Gene_6961	Lema_T119060	lm_SuperContig_22_v2:294086–294450	6654	1114.42	6.32	0.00E+00	Hypothetical protein (AvrLm11)
Gene_11177	Lema_T070880	lm_SuperContig_7_v2:982889–983113	6770	895.39	5.79	0.00E+00	Hypothetical protein (AvrLmJ1)
**11 dpi**							
Gene_9481	Lema_T043000	lm_SuperContig_4_v2:1367322–1368521	2280	179.23	13.5	0.00E+00	Vacuolar protein sorting-associated protein
Gene_10161	Lema_T054900	lm_SuperContig_5_v2:1424583–1425111	1760	173.39	12.26	6.50E-123	Hypothetical protein
Gene_7308	Lema_T122530	lm_SuperContig_25_v2:145516–147997	1128	265.94	10.79	0.00E+00	Glucose dehydrogenase
Gene_2216	Lema_T084480	lm_SuperContig_12_v2:827863–828642	1906	121.93	10.46	1.50E-131	Predicted protein SSP(Effector)
Gene_11875	Lema_T061270	lm_SuperContig_8_v2:1510198–1511697	1484	458.66	10.25	0.00E+00	Peptidase S26A, signal peptidase I/Proteolysis
Gene_2066	Lema_T082980	lm_SuperContig_12_v2:398032–399219	1316	182.72	9.44	1.20E-212	Hypothetical protein
Gene_11099	Lema_T070100	lm_SuperContig_7_v2:754032–755352	2233	136.92	9.05	0.00E+00	Peptidoglycan-binding LysM (Lm5LysM)
Gene_170	Lema_T001700	lm_SuperContig_0_v2:571327–571980	1377	101.99	8.84	0.00E+00	Hypothetical protein (CSEP)
Gene_12054	Lema_T063060	lm_SuperContig_9_v2:365423–366979	1857	106.76	8.42	0.00E+00	3-dehydroquinate synthase activity
Gene_10765	Lema_T049510	lm_SuperContig_6_v2:1209331–1209753	15579	1656.89	8.12	1.40E-127	Hypothetical protein
Gene_616	Lema_T006160	lm_SuperContig_0_v2:2286680–2287371	5059	356.09	7.55	0.00E+00	Hypothetical protein
Gene_10767	Lema_T049530	lm_SuperContig_6_v2:1211326–1212274	2212	253.35	7.23	0.00E+00	Glucose/ribitol dehydrogenase
Gene_1012	Lema_T010120	lm_SuperContig_0_v2:3437723–3438128	1319	310.89	7.13	2.50E-235	Hypothetical protein (CSEP)
Gene_9092	Lema_T039110	lm_SuperContig_4_v2:302019–302430	12896	2333.41	7.08	4.20E-155	Response to stress
Gene_304	Lema_T003040	lm_SuperContig_0_v2:1104438–1107822	1149	150.74	6.8	2.70E-294	Haloacid dehalogenase-like hydrolase
Gene_11976	Lema_T062280	lm_SuperContig_8_v2:1760041–1760879	1061	112.38	6.37	6.70E-263	Fasciclin and related adhesion glycoproteins
Gene_7609	Lema_T025030	lm_SuperContig_2_v2:575802–582958	3787	960.67	6.09	1.10E-83	Hypothetical protein
Gene_6173	Lema_T021470	lm_SuperContig_1_v2:2566440–2566907	1252	141.60	6.06	4.20E-177	Hypothetical protein
Gene_9004	Lema_T038230	lm_SuperContig_3_v2:2336899–2337410	2751	214.78	5.78	0.00E+00	Hypothetical protein (CSEP)
Gene_368	Lema_T003680	lm_SuperContig_0_v2:1246649–1246843	1027	216.39	5.24	4.00E-198	Hypothetical protein (CSEP)
Gene_1613	Lema_T076380	lm_SuperContig_10_v2:1509441–1509941	2195	113.31	5.15	6.30E-275	Hypothetical protein (CSEP)
Gene_3471	Lema_T089840	lm_SuperContig_15_v2:160448–161311	746	150.76	7.37	1.30E-257	Cutinase/catalytic activity

* *The fold change was estimated by comparing in planta gene expression at 3, 5, 7, and 11 days post inoculation (dpi) with axenic cultures of L. maculans*.

*Analyses were performed with five biological replicates. dpi, days post inoculation; FPKM, Fragments per kilobase of transcript per million mapped reads; FC, fold-change; SE, Standard error for FPKM*.

### Expression of CSEPs in *L. maculans* during compatible and incompatible interactions

**Figure 6:**
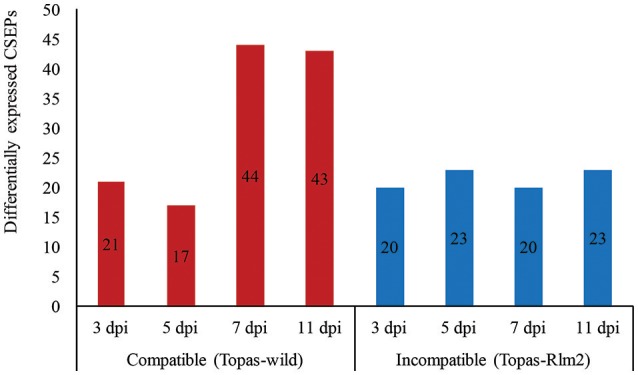
**Number of upregulated candidate small secretory effector proteins (CSEPs) identified by RNA-seq analyses performed in *Leptosphaeria maculans* at different stages of disease development during compatible and incompatible interactions**. Upregulated genes were identified by comparing *in planta* samples at different growth stages with axenic cultures. dpi, days post inoculation. Analyses were performed with five biological replicates and a threshold value of FDR < 0.0001 and log_2_ fold-change > 1.5.

A total of 552 classically secreted proteins were identified using computational pipeline comprising SignalP and TMHMM software tools along with Secretool pipeline. Following further analyses, 134 genes were prioritized as high confidence CSEPs based on the results obtained with EffectorP software. In the case of the compatible interaction with Topas-wild, an important increase in upregulated CSEPs was observed at 7 and 11 dpi, while very limited differences over time were noted in the incompatible interaction (Figure 6).

**Figure 7:**
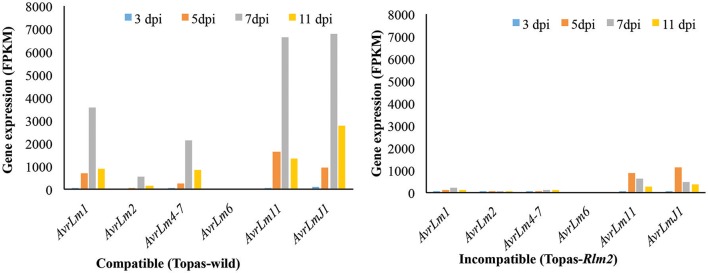
**Expression pattern of known *Avr* genes at different disease developmental stages in *Leptosphaeria maculans* during compatible and incompatible interactions**. FPKM, Fragments per kilo-base of transcript per million mapped reads; dpi, days post inoculation. Analyses were performed with five biological replicates.

Out of the 134 CSEPs, 35 genes were not expressed in either the compatible or incompatible host. However, a total of 28 genes showed the highest expression level at 7 dpi in the compatible interaction, which would link them to the biotrophic phase of the fungus (Table 2). This profile was similar to that of known *Avr* genes, which were mostly found to be highly expressed at 7 dpi in the compatible interaction, with the obvious exception of *AvrLm6* that remained unchanged throughout the sampling periods (Figures 5, 7). The expression patterns of *AvrLm1, AvrLm2, AvrLm4*-7, *AvrLm11*, and *AvrLmJ1* were similar in the compatible interaction as their expression increased over time to reach a peak at 7 dpi, and subsided at 11 dpi. As for the incompatible interaction, limited expression was observed for all known *Avr* genes with no distinctive pattern (Table 2, Figure 7). Of additional significance, an expression pattern similar to *Avr* genes was found for 23 other effectors in the compatible interaction, including eight specific to *L. maculans* and not previously reported as effectors (Table 2). Of these, gene_6114 and gene_2728 are particularly interesting for their differentially higher expression at 7 dpi in Topas-wild. Finally, gene_9004 showed the highest expression at 7 dpi during the compatible interaction at a level of almost 24,000 FPKM.

**Table 2:** **Expression pattern of 28 effector proteins, including known *Avr* genes, during *Leptosphaeria maculans* compatible and incompatible interactions with canola**.

**Gene name**	**Gene ID**	Expression value (FPKM^*^)								***Avr* genes**	**Previously reported**
		**Topas-wild (compatible)**				**Topas-*Rlm2* (incompatible)**					
		**3 dpi**	**5 dpi**	**7 dpi**	**11 dpi**	**3 dpi**	**5 dpi**	**7 dpi**	**11 dpi**		
Gene_9004	Lema_T038230	0.0	122.3	23963.3	2751.1	0.0	106.3	257.6	38.1		1
Gene_1613	Lema_T076380	161.7	2525.2	11944.8	2194.9	79.6	1065.7	1667.9	435.4		1
Gene_1720	Lema_T086760	0.0	299.4	8458.8	2802.2	7.9	744.5	1618.6	1428.4		1
Gene_11177	Lema_T070880	77.5	928.2	6769.7	2786.3	10.6	106.3	487.2	375.2	*AvrLmJ1*	1.2
Gene_6961	Lema_T119060	51.0	1646.3	6653.6	1346.6	32.0	899.8	627.7	263.3	*AvrLm11*	2
Gene_8487	Lema_T033060	82.9	1626.7	5468.1	728.6	91.9	463.7	322.1	58.5		1
Gene_12203	Lema_T064550	361.8	939.2	4087.3	3933.3	350.5	1046.2	470.3	385.3		
Gene_2817	Lema_T081180	32.0	32.3	3930.2	1420.1	30.6	354.6	679.8	164.6		1
Gene_6375	Lema_T113200	67.4	1117.1	3555.8	959.4	23.7	437.1	627.4	188.6		1
Gene_10780	Lema_T049660	57.3	673.4	3553.0	875.5	29.0	108.0	205.8	134.1	*AvrLm1*	2
Gene_7223	Lema_T121680	61.1	448.7	2982.2	1139.6	71.0	323.9	211.3	291.7		1
Gene_6114	Lema_T020880	14.5	937.1	2479.8	722.8	14.4	145.6	8.9	71.3		1
Gene_2397	Lema_T086290	23.6	237.1	2130.8	859.7	18.8	64.8	134.7	116.7	*AvrLm4*-7	2
Gene_7362	Lema_T123070	0.0	87.8	1952.3	412.2	0.0	232.6	93.3	308.9		2
Gene_1698	Lema_T086540	15.8	411.8	1775.8	607.6	18.6	230.1	110.7	118.2		1
Gene_170	Lema_T001700	0.0	41.2	1695.1	1377.3	17.5	297.1	289.5	150.6		
Gene_6860	Lema_T118050	385.1	636.1	1551.7	918.3	325.5	351.1	233.1	192.1		1
Gene_2728	Lema_T080290	0.5	1.6	804.9	112.6	17.7	49.6	124.6	141.4		
Gene_5357	Lema_T013310	225.6	210.0	574.7	236.9	137.6	65.9	43.7	21.3		
Gene_10809	Lema_T049950	0.0	62.8	526.2	154.7	5.2	12.4	20.3	20.2	*AvrLm2*	1
Gene_8681	Lema_T035000	0.0	296.1	466	56.8	0.0	75.9	83.1	157.4		1
Gene_7202	Lema_T121470	19.3	116.8	453.4	211.5	7.3	45.3	22.4	49.2		
Gene_7203	Lema_T121480	0.0	85.6	435.3	97.3	0.0	18.0	59.7	0.0		1.2
Gene_3097	Lema_T095640	48.1	40.4	369.8	155.8	59.6	219.7	185.9	66.1		1
Gene_11572	Lema_T058240	0.0	0.0	300.2	3.7	0.0	17.5	225.4	0.0		
Gene_9602	Lema_T044210	0.0	56.1	136.2	54.6	0.0	19.9	3.3	1.1		1
Gene_11574	Lema_T058260	0.0	0.0	130.6	2.9	0.0	0.0	110.4	16.6		
Gene_11156	Lema_T070670	57.9	30.8	89.1	25.1	22.5	5.3	3.5	9.2		

*RNA-seq analysis was performed for L. maculans isolate D5 inoculated to Topas-wild (compatible) and Topas-Rlm2 (incompatible) canola genotypes*.

* *Analyses were performed with five biological replicates. dpi, days post inoculation; FPKM, Fragments per kilo-base of transcript per million mapped reads; 1, (Haddadi et al., 2016); 2, (Lowe et al., 2014)*.

In addition to the effectors listed in Table 2, 15 other genes identified as CSEPs showed higher levels of expression exclusively during the compatible interaction at 11 dpi when the fungus had entered its necrotrophic phase (Table 3). Furthermore, the crinkler effector search performed initially in *P. sojae* identified exactly the same set of proteins reported earlier by Haas et al. (2009) (Supplementary Table 3). This validated the method used here to identify crinkler effectors in *L. maculans* genome. The initial search identified 63 proteins with LFLAK-like domain present within the initial 100 AA (Supplementary Table 4). Further analysis with secretool revealed only five proteins and EffectorP confirmed only one candidate (Gene_2728, Lema_T080290.1) as a crinkler effector (Gene_2728, Lema_T080290.1). Gene_2728 showed the highest expression at 7 dpi during the compatible interaction (Supplementary Figure 2).

**Table 3:** **Expression pattern of 15 effector proteins highly expressed during the necrotrophic phase of a compatible interaction of *Leptosphaeria maculans* with canola**.

**Gene name**	**Gene ID**	Expression value (FPKM^*^)							
		**Topas-wild (compatible)**				**Topas-*Rlm2* (incompatible)**			
		**3 dpi**	**5 dpi**	**7 dpi**	**11 dpi**	**3 dpi**	**5 dpi**	**7 dpi**	**11 dpi**
Gene_8419	Lema_T124480	62.8	21.6	230.5	4859.8	47.2	112.1	409.5	223.9
Gene_6367	Lema_T023410	0.0	130.8	611.8	2132.9	46.1	265.2	325.7	184.8
Gene_368	Lema_T003680	237.6	357.1	74.3	1026.7	215.5	243.9	0.0	318.7
Gene_3948	Lema_T103880	0.0	0.0	36.6	829.0	20.0	0.0	110.7	2.2
Gene_4618	Lema_T102900	1.0	0.0	197.2	338.5	1.2	0.0	0.0	89.5
Gene_8514	Lema_T033330	8.5	11.1	34.2	258.5	9.5	201.0	72.1	150.0
Gene_2889	Lema_T081900	0.0	11.6	16.7	224.8	0.0	50.0	0.0	4.4
Gene_11386	Lema_T056380	19.0	15.1	156.2	202.1	37.9	21.1	34.0	16.1
Gene_776	Lema_T007760	54.7	55.9	45.3	178.5	44.6	109.6	60.7	90.8
Gene_387	Lema_T003870	7.3	21.3	99.0	174.5	43.8	72.1	44.1	85.2
Gene_775	Lema_T007750	37.0	0.0	32.6	99.1	31.9	49.7	65.1	34.4
Gene_588	Lema_T005880	16.7	25.2	67.5	97.2	24.8	31.7	10.0	4.5
Gene_1689	Lema_T077140	16.6	6.2	26.3	56.8	25.2	34.7	8.6	13.7
Gene_8091	Lema_T029850	27.5	4.4	27.4	45.4	28.4	4.2	27.1	4.5
Gene_4923	Lema_T109480	13.0	4.7	57.3	61.9	9.9	6.3	32.3	3.1

* *Analyses were performed with five biological replicates. dpi, days post inoculation; FPKM, Fragments per kilo-base of transcript per million mapped reads*.

### Transcription factors associated with effector expression

**Figure 8:**
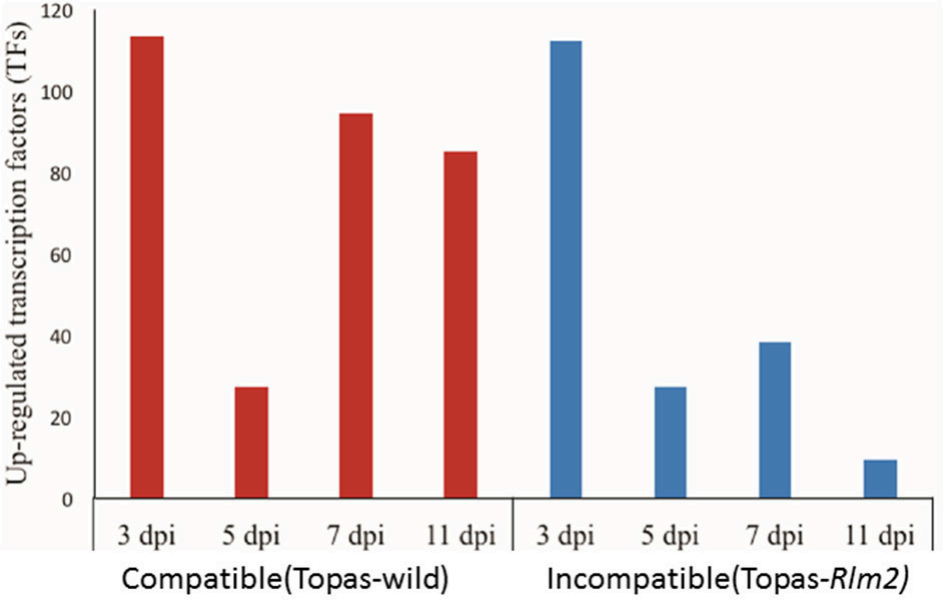
**Number of transcription factors (TFs) upregulated in *Leptosphaeria maculans* at 3, 5, 7, and 11 days post inoculation (dpi) on compatible host Topas-wild and incompatible host Topas*-Rlm2***. Upregulated genes were identified by comparing *in planta* samples at different growth stages with axenic cultures. Analyses were performed with five biological replicates and a threshold value of FDR < 0.0001 and log_2_ fold-change > 1.5.

TFs regulate the expression of a number of genes simultaneously, and their upregulation during *in vivo* conditions was indicative of their importance in the outcome of the interaction. Both compatible and incompatible interactions were regulated by a large number of upregulated TFs in *L. maculans* in the early events (3 dpi) followed by a sharp decline at 5 dpi (Figure 8). As with the phenotypes, a clear distinction between compatible and incompatible interactions appeared at 7 dpi where nearly 100 TFs were upregulated in the former case compared to 40 in the latter, and these differences were even more manifest at 11 dpi (Supplementary Table 5). Among TFs, the LmStuA TF (gene_1191), a member of APSES domain containing proteins was found to be strongly upregulated at 7 dpi in the compatible interaction with Topas-wild, thus suggesting its importance in the infection process. Mostly Zn2Cys6 TFs were upregulated during the compatible interaction (Supplementary Table 5).

### Expression of CAZymes in *L. maculans* during compatible and incompatible interactions

**Figure 9:**
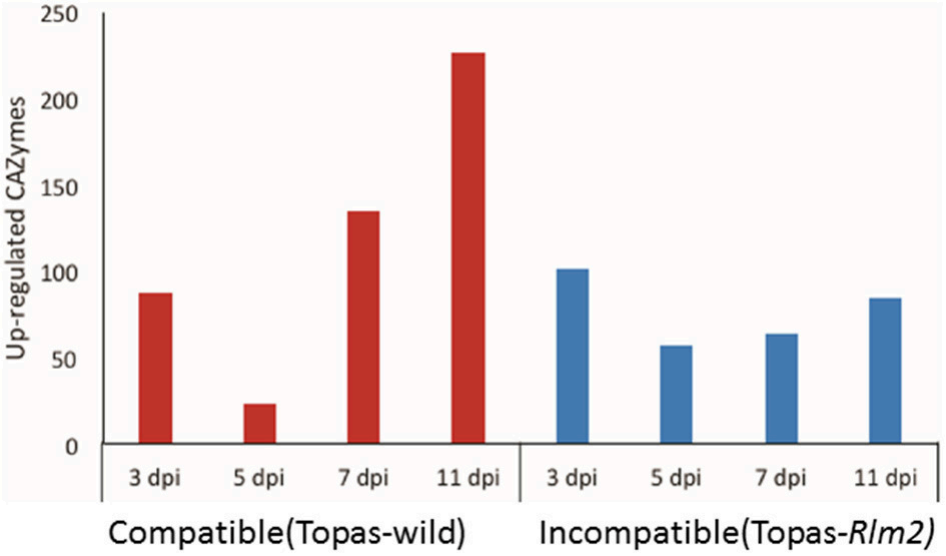
**Number of carbohydrate active enzymes (CAZymes) upregulated at 3, 5, 7, and 11 days post inoculation (dpi) of *Leptosphaeria maculans* inoculated to compatible host Topas-wild and in-compatible host Topas*-Rlm2***. Upregulated genes were identified by comparing *in planta* samples with axenic cultures. Analyses were performed with five biological replicates and a threshold value of FDR < 0.0001 and log_2_ fold-change > 1.5.

Plant-fungal interactions involve a variety of CAZymes that are used by the fungal pathogen to infect its host. RNA-seq data for *L. maculans* infecting compatible host Topas-wild and incompatible host Topas-*Rlm2* showed a high number of differentially expressed CAZymes at 3 dpi (Figure 9). At 5 dpi, this number dropped drastically. It remained fairly constant thereafter in the case of the incompatible interaction (Figure 9). By contrast, we observed a rapid increase in differentially expressed CAZymes from 5 to 7 dpi in the compatible interaction, and this number exceeded 200 at 11 dpi, supporting their role in the necrotrophic phase of the fungus.

Among the differentially expressed CAZymes, glycosyl hydrolase (GH), carbohydrate esterase (CE) domain containing genes were more prevalent followed by those belonging to auxiliary activities (AAs) class (Supplementary Table 6). During the early stages (3 dpi) of *L. maculans* infection, cellulose and pectin-degrading enzymes such as PL3, CE4, GH43, and GH3 were upregulated in both interactions compared to axenic growth. At the later stage (11 dpi), CE1, PL1, GT34, and GH28 were only upregulated in the compatible interaction. Carbohydrate binding molecules (CBM) were another prevalent group among DEGs. In *L. maculans*, enzymes with LysM motifs are a well-studied class of CAZymes. Among the LysM domain containing genes present in *L. maculans* genome, *Lm2LysM* (Gene_4592) and *Lm5LysM* (Gene_11099) genes showed a higher level of expression at 7 dpi during the compatible interaction (Supplementary Figure 3). However, *Lm4LysM* (Gene_7646) gene was not expressed during either the compatible or incompatible interaction. The expression pattern for some of the CAZymes at different growth stages of *L. maculans* during compatible and incompatible interaction is shown in Supplementary Figure 4.

### Expression of important peptidases, secondary metabolites, and necrosis-inducing proteins (NIPs)

**Figure 10:**
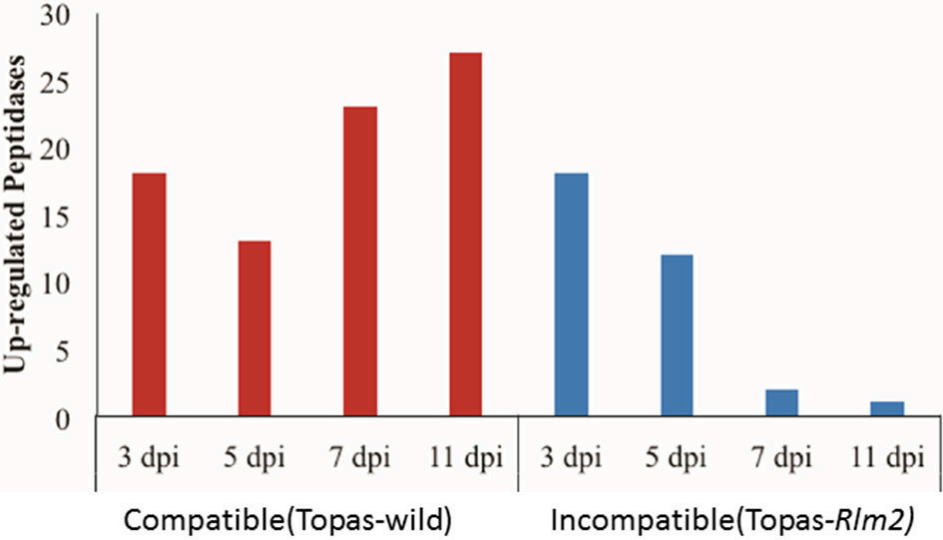
**Number of upregulated peptidases observed at 3, 5, 7, and 11 days post inoculation (dpi) of *Leptosphaeria maculans* inoculated to compatible host Topas-wild and in-compatible host Topas*-Rlm2***. Upregulated genes were identified by comparing *in planta* samples with axenic cultures. Analyses were performed with five biological replicates and a threshold value of FDR < 0.0001 and log_2_ fold-change > 1.5.

**Figure 11:**
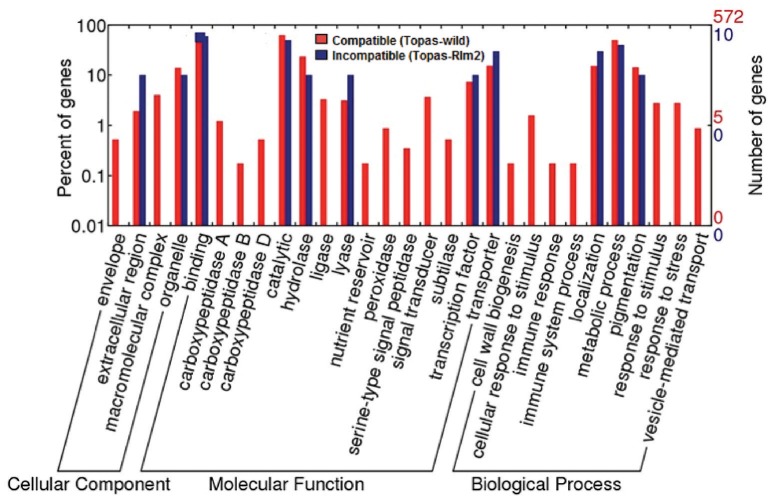
**Functional categorization of upregulated genes at 7 days post inoculation (dpi) during compatible and incompatible canola-*Leptosphaeria maculans* interactions**. The right y-axis indicates the number of genes in a category. The left y-axis indicates the percentage of a specific category of genes in the main category. Upregulated genes were identified by comparing *in planta* samples with axenic cultures. Analyses were performed with five biological replicates and a threshold value of FDR < 0.0001 and log_2_ fold-change > 1.5.

At the early stages of infection, a similar number of differentially expressed peptidases were observed in both interactions. However, as the fungus transitioned from the biotrophic phase at 7 dpi to the necrotrophic phase at 11 dpi, the difference in the number of differentially expressed peptidases between the compatible and incompatible interaction steadily increases to a ratio of over 60 to 1 at 11 dpi (Figure 10, Supplementary Table 7). Among the different classes of peptidases, serine proteases and carboxypeptidases were highly upregulated during *L. maculans* compatible interaction (Figure 11, Supplementary Table 7).

In *L. maculans*, a cluster of 23 genes including PKS, NRPs genes and 18 Sir genes have been identified (Gardiner et al., 2004). Most of these genes showed a similar pattern of expression at all the stages of infection with a higher expression at 11 dpi compared to early growth stages of *L. maculans in planta* (Supplementary Figure 5A). By contrast, Sir O (oxidoreductase) and Sir T (thioredoxin reductase) showed higher level of expression at both 3 dpi and 11 dpi (Supplementary Figure 5A). However, comparative analyses indicated that there was no difference in expression at 3 dpi between the interactions (Supplementary Figure 5B). Most of the NRPs showed high level of expression at 3 dpi and 11 dpi but NRP gene_904 showed high expression at 7 dpi (FPKM 876) that receded at 11 dpi (FPKM 112.2). For the two necrosis-and ethylene inducing proteins (Neps) in *L. maculans*, Nep1-like proteins (NPP1, gene_11090) showed higher expression during the pathogenic phases of the fungus (Supplementary Table 8).

### Functional categorization and gene ontology (GO) enrichment of the DEGs

Differential molecular responses in *L. maculans* under compatible and incompatible interactions were observed based on the functional categorization of DEGs (Figure 11, Supplementary Figure 6). The DEGs were grouped into three main classes, molecular function, cellular component, and biological process, and several subclasses based on gene ontology terms assigned using WEGO tool. During early asymptomatic stage at 3 dpi, upregulated genes with similar functional categories were observed under both conditions (Supplementary Figure 6). However, the molecular responses of *L. maculans* between the compatible and incompatible hosts differed significantly over time, and were more prominent at 7 and 11 dpi (Figure 11; Supplementary Figure 6). The most striking differences were found in the number of genes expressed in each sub-category and in the prevalence of functional categories linked to a transition between the biotrophic and necrotrophic phases such as carboxypeptidases, ligase, peroxidases, cell-wall biogenesis, and stimuli (Figure 11).

Hierarchical clustering and GO enrichment of DEGs during the compatible interaction was also performed using AgriGO tool. Most of the DEGs were found to belong to binding and catalytic functions (Supplementary Figure 7). Localization (GO:0051179, 3.21e-62), cellular process (GO:0009987, 2.37e-258), signal transduction (GO:0007165, 3.48e-13), response to stress (GO:0006950, 8.44e-11), and transcription regulator activity (GO:0030528, 3.3e-15), were the most significantly enriched GO terms at 3 and 5 dpi. At 7 dpi, localization (GO:0051179, 2.35e-61), transport (GO:0006810, 1.68e-61), catabolic process (GO:0009056, 1.03e-11), metabolic process (GO:0008152, 0), gene expression (GO:0010467, 3.93e-38), binding (GO:0005488, 6.84e-319), and transferase activity (GO:0016741, 8.4e-09) were linked to the highest number of genes, and at 11 dpi, hydrolytic activity, mostly monooxygenase activity (GO:0051179, 5.86e-09), transcription regulator activity (GO:0030528, 2.49e-21), transcription factor activity (GO:0051179, 3.14e-14), and regulation of biological process (GO:0050789, 7.09e-26) were the most common enriched GO terms (Supplementary Figure 7).

## Discussion

In the present study, we performed a comprehensive analysis of the *L. maculans* transcriptome profile during compatible and incompatible interactions with canola. Based on comparative analyses, key genes that dictate both the interaction between canola and *L. maculans* and the different pathogenic stages of the fungus were highlighted. Among the genes of particular significance, our results have identified candidate effectors, TFs, CAZymes, peptidases, and other pathogenesis-related genes that are specifically upregulated as *L. maculans* initiates a biotrophic interaction with the plant, and transitions to a necrotrophic phase. The differential expression of genes during compatible and incompatible interactions offers a precise insight into the mechanisms of pathogenesis in the *L. maculans*-canola interactions.

The effectiveness of transcriptome analyses in plant-pathogen interactions depends primarily on the approach of expression quantification, statistical methods avoiding possible errors and reliance on normalized comparisons, experimental design with sufficient replications and appropriate plant or pathogen material to address relevant biological questions (Williams et al., 2014). In this work, introgression lines Topas-wild and Topas*-Rlm2* were used to study *L. maculans* molecular responses during disease development. Topas-wild is a common cultivar from Canada well known and exploited for its susceptibility to *L. maculans* isolate D5 (Larkan et al., 2013). On the other hand, Topas*-Rlm2* is a recently developed cultivar that carries *Rlm2*, a major resistance gene that prevents infection from *L. maculans* isolate D5 (Larkan et al., 2015). As such, this cultivar provided a unique opportunity to investigate the subtle elements that distinguish the ability of *L. maculans* to infect or not its host. The importance of obtaining good reproducible phenotypes for transcriptomic analyses cannot be overstated as it remains the reference basis for all analyses. On the basis of visual observations, this condition was clearly met as Topas-wild plants exhibited clear symptoms of infection over the course of the experiment that culminated with the presence of extensive necrotic tissues at 11 dpi while the infection never extended beyond the point of inoculation in Topas*-Rlm2* plants. Other studies have reported similar symptom progression at varying time points (Lowe et al., 2014; Haddadi et al., 2016), thus suggesting that experimental conditions can influence the rapidity with which *L. maculans* can infect its host. For this reason, it is critical to ensure that sampling procedures include enough replications that will capture an accurate biological variability within a condition. In this work we have used five biological replications to enhance both the biological and statistical power to compare gene expression across stages and conditions. We have also collected the whole infected cotyledons for analysis to achieve a thorough understanding of pathogen transcriptome activities at cotyledon level during infection. This approach was validated by our PC analysis (see Figure 3) where the clustering of samples confirmed a uniformity within a given treatment and a variability among treatments thereby supporting that observed phenotypes were indeed associated with differential gene expression. This further supported subsequent statistical analyses of DEGs.

### Expression of known *avrs* and CSEPs during biotrophic and necrotrophic phases

Several *Avr* and *R* genes have been identified and/or proposed to play a role in the *L. maculans*-canola interaction (Balesdent et al., 2002; Ghanbarnia et al., 2012). Following our analyses, all known *Avr* genes observed by Lowe et al. (2014) were also found to be highly expressed at 7 dpi, and reduced in their expression at 11 dpi in the compatible interaction. Based on a comparison with Topas*-Rlm2*, it is apparent that the period 5–7 dpi harbors the biotrophic phase of *L. maculans* and 11 dpi is more consistent with the necrotrophic phase. As such the differential expression of *Avrs* at 7 dpi indicates that some of them are clearly involved primarily in the establishment of biotrophy, and possibly the transition to necrotrophy but are no longer relevant when *L. maculans* has entered its necrotrophic phase.

With the availability of full genome sequences and more advanced computational tools and pipelines, we were able to identify and characterize effectors in *L. maculans* and better address their potential role/functionality. From strict computational predictions, there are 552 classically secreted proteins in *L. maculans*, which represents an unrealistic number in terms of functional effectors (Sonah et al., 2016). Recently developed tools based on machine learning were used here to further prioritize 134 CSEPs that showed evidence of upregulation in RNA-seq data. At 3 dpi, which corresponded to an asymptomatic growth phase, a common set of effectors highly expressed in both the compatible and incompatible conditions was observed, which suggests they play a minimal role in the fate of the interaction. By contrast, the expression pattern of effectors varied drastically between the compatible and incompatible conditions at 7 dpi. This approach allowed to narrow down the list of possible functional effectors to 28 that were uniquely upregulated at 7 dpi only under compatible conditions and thus presumed to be important for the establishment and maintenance of the biotrophic phase. As a matter of fact, many of the identified effectors here were either known *Avr*, or genes for which a role in pathogenicity was suggested (Haddadi et al., 2016). In addition, eight new effectors are proposed on the basis of their features and expression, and should be interesting candidates in future functionality assays. The studies by Haddadi et al. (2016) and Lowe et al. (2014) used different strains of *L. maculans*, which explains the differences in the number of differentially expressed effectors. For instance, *AvrLm6* was not expressed in our study since the *L. maculans* strain D5 does not have *AvrLm6*. This suggests that it should be important to properly assess resistant germplasm with specific *L. maculans* strains present in a given region.

Compared to biotrophic effectors, very few effectors responsible for the necrotrophic phase are known (Lo Presti et al., 2015). In the present study, on the basis of comparative expression in compatible and incompatible interactions, we have identified 15 effectors that were distinct from *Avr* genes (and associated effectors) in their chronology of expression. This suggests that these effectors are specific to the necrotrophic phase of the fungus and that *L. maculans*, as a hemibiotroph, has indeed evolved different mechanisms to support its biotrophic and necrotrophic phase. At the same time, high expression of a single crinkler-type effector at 7 dpi in *L. maculans* suggests its involvement in the transition from a biotrophic to a necrotrophic phase. However, the occurrence of a single crinkler in *L. maculans* compared to 74 in *P. sojae* suggests that *L. maculans* does not have the crinkler-mediated mechanism leading to the necrotrophic phase found in *P. sojae* and other oomycetes.

A biotrophic-necrotrophic effector system that has been well studied is that of *Phytophthora infestans* in its interaction with potato (Whisson et al., 2007). During the biotrophic phase, *P. infestans* secretes AVR3a from its haustoria to suppress cell-death; as the oomycete moves to a necrotrophic stage, *AVR3a* is downregulated (Whisson et al., 2007). Similarly, our results showed that all 28 genes, including known *Avr* genes, were highly expressed during the biotrophic phase at 7 dpi and their expression reduced during the necrotrophic phase at 11 dpi. The same genes showed limited or no expression in the incompatible interaction. This suggests that these genes produce functional effectors and are key factors responsible for cross-talk between *L. maculans* and its host. Another gene found here, *Lm5LysM*, is highly expressed at 7 dpi and shows homology with the *SLP1* gene of *M. grisea* that is expressed at the interface between the fungal cell wall and host cell plasma membrane during biotrophic invasion. The LysM-type effectors have been previously associated with biotrophy and shown to be involved in plant-fungus interactions (Gust et al., 2012; Kombrink and Thomma, 2013; Lowe et al., 2014).

### Expression pattern of NLPs confirming the necrotrophic stage in *L. maculans*

In the present study, the highest level of expression for Lm-NLP (gene_11090) gene at 11 dpi, as *L. maculans* entered into its necrotrophic phase, is an observation consistent with the results of Haddadi et al. (2016). Indeed, these authors used the NLP gene expression profile as a means to distinguish genes related to the biotrophic or necrotrophic phase in *L. maculans*; the induction of necrosis by Lm-NLP was confirmed with a transient assay in tobacco (Haddadi et al., 2016). Our results do confirm that the highest expression of NLPs is synchronized with the necrotrophic phase, while that of known *Avrs* with the biotrophic phase. *Avrs* and *NLPs* can thus be considered as valid markers to characterize CSEPs as biotrophic or necrotrophic effectors. Similar findings were observed in *P. infestans* where INF1 and Nep1-like effectors are secreted at later stages of infection that correspond to *P. infestans* transitioning from a biotrophic to a necrotrophic stage (Kanneganti et al., 2006).

### Expression dynamics of CAZymes and TFs

The increasing number of upregulated CAZymes from 5 to 11 dpi in the compatible interaction can be related to the biphasic life style of *L. maculans* as previously suggested by Lowe et al. (2014). This is particularly relevant when compared to the incompatible interaction, where this number remained relatively unchanged over the course of infection. Our results showed that GH, CE, and AA were the most prevalent groups of CAZymes among upregulated genes. During the early stages (3 dpi) of *L. maculans* infection, mostly cellulose and pectin-*degrading* enzymes were the most prevalent groups of CAZymes among upregulated genes. On the other hand, GH, CE and AA families were predominantly upregulated during the necrotrophic stage. Pathogenic fungi face the plant cell wall as a first barrier to establish infection. Plant cell walls are mainly composed of carbohydrates and glycoproteins. To breakdown this barrier, plant pathogenic fungi need to secrete a diverse range of carbohydrate-active enzymes (CAZymes). However, our results clearly showed that the release of CAZymes in the early stages is not a key determinant of the interaction since their number and expression were similar in both interactions.

CAZymes are also involved in nutrient uptake and those prominently expressed at later stages of infection are thought to be involved in uptake of amino acid and sugars from the host. Our results bring stronger support to the concept suggested by Lowe et al. (2014) that CAZymes play an important role in *L. maculans*-canola interaction, notably during the establishment of necrotrophy.

Biphasic expression turnover similar to that of CAZymes was also observed with TFs. As the infection progressed in the compatible interaction, a much higher number of differentially expressed TFs was noted especially from 5 to 7 dpi. This period is critical in the fate of the interaction since it is clearly synchronized with the establishment of the infection in the compatible interaction. Of particular importance, the LmStuA TF, a member of APSES domain-containing proteins, showed its highest expression at 7 dpi in *L. maculans* interaction with Topas-wild. The expression profile of APSES domain-containing genes observed in this study is well aligned with an earlier report by Soyer et al. (2015). The StuA TF was found to be involved in morphogenesis, metabolites production and effector regulation (Baeza-Montañez et al., 2015; Soyer et al., 2015). Recently, StuA was suggested to play a key role in the *L. maculans*-canola interaction since its silencing led to a reduced expression of *AvrLm1, AvrLm6*, and *AvrLm4*-7, at 7dpi (Soyer et al., 2015). Moreover, the Zn2Cys6 TFs were upregulated in the compatible interaction supporting its role in regulating pathogenicity-related genes during the biotrophic phase. The Zn2Cys6 TFs have been reported to be involved in different regulatory functions. They are also unique to fungi and have been extensively studied in *Saccharomyces cerevisiae* and *Aspergillus nidulans* (Shimizu et al., 2003; Vienken et al., 2005).

### Secondary metabolites involved in the pathogenesis of *L. maculans*

Secondary metabolites like NRPs and phytotoxins such as sirodesmin were found to have a differential expression pattern between the compatible and incompatible interactions. Differences in gene expression pattern between the compatible and incompatible interaction were observed for NRPs, known to be involved in the production of phytotoxins, siderophores, and pigments, mostly at 11 dpi. Another important phytotoxin involved in the establishment of necrosis is sirodesmin, which is regulated by Sir genes (Gardiner et al., 2004; Haddadi et al., 2016). Most Sir genes were found to be highly expressed at 11 dpi compared to *in planta L. maculans* early growth stages in this study. A notable exception was SirO and SirT, which were highly expressed at 3 dpi and are thought to be involved in the production of sirodesmin early in the infection process. Indeed, Gardiner et al. (2004) have also reported significant amount of sirodesmin production at 4 dpi with *L. maculans*. On the other hand, the fact that SirT was equally expressed in the compatible and incompatible interactions at 3 dpi in our work would suggest that additional factors must complement their activity for infection to occur.

Based on our WEGO functional annotation, the respective vigorous and restricted growth of *L. maculans* on the compatible and incompatible hosts correlated very well with enrichment of genes in functional categories. Toward the later stages of the experiment, very few classes of functional categories were represented in the incompatible interaction, an observation that clearly confirms the inability of *L. maculans* to infect Topas*-Rlm2*. However, it is not possible to conduct robust statistical analyses because of the disparity in the number of genes between the compatible (572) and incompatible interactions (10). Nevertheless, these analyses give a good qualitative visualization of the genes and functions, and their relative importance in the case of compatibility. Interestingly, these analyses also highlighted in the compatible interaction how specific functional classes were indicative of the biotrophic or necrotrophic stage, and the transition from one to the other.

Validation of DEGs identified with transcriptome profiling is always a concern to build a confidence about the significance of results. The validation of DEGs by qPCR is expected for the microarray studies or transcriptomic studies with no or limited biological replications (Deshmukh et al., 2010; Xie et al., 2016). For instance, microarray studies mostly relied on qPCR to validate the results since there is a limit for the number of probes hybridized to the target in microarrays. Therefore, the highest level of gene expression is never truly represented for highly expressed genes. This also leads to a bias of data normalization and reduced linear range. By contrast, RNA-seq provides digital expression for the entire transcripts without any minimum or maximum count limit. In our study, we have used five replications, in line with the robust standards accepted for RNA-seq experiments (Fang and Cui, 2011). This is further supported by the fact that all previously identified Avr genes (*AvrLm1, AvrLm4*-7, *AvrLm11, AvrLmJ1, AvrLm2*) had an expression pattern consistent with expectations and previous reports (Lowe et al., 2014; Haddadi et al., 2016).

## Conclusions

Hemibiotrophic fungi such as *L. maculans* have a distinctive life style that involves complex molecular processes and the expression turnover of thousands of genes. The atlas of gene expression provided here will be helpful to understand the molecular crosstalk between canola and *L. maculans* as it relates to compatibility or incompatibility, and could be exploited toward the deployment of novel strategies to overcome blackleg disease. The comparison made between the compatible and incompatible interaction highlighted the role of specific CSEPs, CAZymes, TFs, and secondary metabolites involved in the infection process. The differential expression pattern observed for these classes of pathogenicity-related genes can serve as a valuable resource to differentiate compatible and incompatible interactions in the context of developing resistant canola germplasm. In addition, our time series analysis based on a comprehensive and comparative differential gene expression as *L. maculans* infects a susceptible or resistant host has brought to light elements that define the biotrophic and necrotrophic phases of the fungus, as well as some of the mechanisms involved in the transition between the two phases.

## Author contributions

HS performed RNA extractions, library preparations, data analysis, and compilation; XZ and WF performed all bio-assays, maintained the fungal cultures, and performed RNA extractions, HB developed introgression line and provided seed material, RD and RB contributed to data analysis and overall conclusions, HS and RB wrote initial draft of the MS with final contributions from XZ, RD, HB, and WF. RB designed and directed the project.

## Funding

This study is funded by the Agri-Innovation program Growing Forward 2, SaskCanola and Agriculture and Agri-Food Canada, and the Canada Research Chair in plant protection to RB.

### Conflict of interest statement

The authors declare that the research was conducted in the absence of any commercial or financial relationships that could be construed as a potential conflict of interest.
