# Supplementary material for: Comparative Transcriptomic Analysis of Virulence Factors in Leptosphaeria maculans during Compatible and Incompatible Interactions with Canola
Source: Front Plant Sci. 2016 Dec 1;7:1784. doi: 10.3389/fpls.2016.01784 (PMC5131014; doi:10.3389/fpls.2016.01784)
Supplement: Supplementary file 1 [file Table1.DOCX]

**Supplementary Table 1.** Summary of transcriptome sequencing performed for *Leptosphaeria maculans* inoculated to canola compatible host Topas-wild and incompatible host Topas*-Rlm2*. The raw reads were obtained with Hiseq sequencer, processed and mapped to the *L. maculans* genome.

| **Growth stage** | **Raw reads** | **Processed Reads** | **Mapped reads** | **Mapped reads (%)** |
| --- | --- | --- | --- | --- |
| *L. maculans* axenic culture | 35098557 | 35036043 | 32676997 | 93.27 |
| *L. maculans* on Topas-Wild | | | | |
| *In planta* at 3dpi | 35196577 | 35155600 | 146676 | 0.42 |
| *In planta* at 5dpi | 31235598 | 31196437 | 155523 | 0.50 |
| *In planta* at 7dpi | 30045932 | 30012477 | 330870 | 1.10 |
| *In planta* at 11dpi | 32270605 | 32224949 | 3838444 | 11.91 |
| *L. maculans* on Topas*-Rlm2* | | | | |
| *In planta* at 3dpi | 38132444 | 38093218 | 130368 | 0.34 |
| *In planta* at 5dpi | 35121228 | 35079208 | 101026 | 0.26 |
| *In planta* at 7dpi | 29711783 | 29682010 | 101015 | 0.36 |
| *In planta* at 11dpi | 32488364 | 34200667 | 209184 | 0.73 |

The data presented is an average of five biological replicates
