# Supplementary material for: Comparative Transcriptomic Analysis of Virulence Factors in Leptosphaeria maculans during Compatible and Incompatible Interactions with Canola
Source: Front Plant Sci. 2016 Dec 1;7:1784. doi: 10.3389/fpls.2016.01784 (PMC5131014; doi:10.3389/fpls.2016.01784)
Supplement: Supplementary file 2 [file Table2.DOCX]

**Supplementary Table 2**. List of 20 highly expressed upregulated genes at early, mid and late in planta growth stages of *Leptosphaeria* *maculans* during incompatible interaction with Topas-*Rlm2*.

| **Gene Name** | **Gene ID** | **Locus** | **FPKM** | **Log2 (FC)** | **Annotation** |
| --- | --- | --- | --- | --- | --- |
| **3dpi** | | | | | |
| Gene_11912 | Lema_T061640 | lm_SuperContig_8_v2:1610355-1610701 | 10812.30 | 12.78 | Hypothetical protein |
| Gene_9702 | Lema_T050310 | lm_SuperContig_5_v2:114784-120578 | 1215.63 | 10.48 | Chitin binding |
| Gene_4884 | Lema_T109090 | lm_SuperContig_18_v2:862571-862990 | 9817.18 | 10.22 | Hypothetical protein |
| Gene_11624 | Lema_T058760 | lm_SuperContig_8_v2:828284-829346 | 2127.14 | 10.10 | Hypothetical protein  SSP(Effector) |
| Gene_1799 | Lema_T087550 | lm_SuperContig_11_v2:702412-703074 | 7226.25 | 9.98 | Hypothetical protein  CSEP |
| Gene_9395 | Lema_T042140 | lm_SuperContig_4_v2:1136979-1138783 | 2787.55 | 9.94 | Alcohol dehydrogenase |
| Gene_6862 | Lema_T118070 | lm_SuperContig_21_v2:752639-754522 | 1076.04 | 9.63 | G-protein coupled receptor activity |
| Gene_2936 | Lema_T094030 | lm_SuperContig_14_v2:218210-219154 | 1455.97 | 9.61 | Heterochromatin-associated protein HP1 |
| Gene_6479 | Lema_T114240 | lm_SuperContig_20_v2:523704-524501 | 5643.29 | 8.34 | 3'-5'-exoribonuclease activity |
| Gene_8982 | Lema_T038010 | lm_SuperContig_3_v2:2203740-2205537 | 1146.61 | 8.04 | Transporter activity |
| Gene_11976 | Lema_T062280 | lm_SuperContig_8_v2:1760041-1760879 | 2957.04 | 7.86 | Fasciclin and related adhesion glycoproteins |
| Gene_7649 | Lema_T025430 | lm_SuperContig_2_v2:804099-805458 | 1288.01 | 7.70 | Predicted transporter |
| Gene_11804 | Lema_T060560 | lm_SuperContig_8_v2:1317923-1319474 | 1049.95 | 7.50 | Hypothetical protein  SSP(Effector) |
| Gene_5070 | Lema_T110950 | lm_SuperContig_19_v2:299282-300301 | 1658.26 | 7.43 | Hypothetical protein |
| Gene_3811 | Lema_T093240 | lm_SuperContig_15_v2:1293850-1295649 | 1782.64 | 7.08 | Transporter activity |
| Gene_1331 | Lema_T073560 | lm_SuperContig_10_v2:445682-445936 | 1448.00 | 6.50 | Hypothetical protein |
| Gene_2652 | Lema_T079530 | lm_SuperContig_13_v2:633177-634702 | 2627.15 | 6.18 | Hypothetical protein |
| Gene_5572 | Lema_T015460 | lm_SuperContig_1_v2:975571-976381 | 1349.67 | 6.18 | Hypothetical protein |
| Gene_1670 | Lema_T076950 | lm_SuperContig_10_v2:1636789-1637830 | 1312.73 | 6.16 | Hypothetical protein |
| Gene_6173 | Lema_T021470 | lm_SuperContig_1_v2:2566440-2566907 | 1210.15 | 6.01 | Hypothetical protein |
| Gene_2949 | Lema_T094160 | lm_SuperContig_14_v2:245949-249071 | 2187.56 | 5.84 | Transport protein |
| **5dpi** | | | | | |
| Gene_4884 | Lema_T109090 | lm_SuperContig_18_v2:862571-862990 | 30829.10 | 12.62 | Hypothetical protein |
| Gene_10273 | Lema_T056020 | lm_SuperContig_5_v2:1712896-1714128 | 1595.48 | 11.45 | Runt and related transcription factors |
| Gene_8310 | Lema_T032040 | lm_SuperContig_2_v2:2602620-2603501 | 1514.60 | 11.45 | Oxidoreductase activity |
| Gene_1171 | Lema_T011710 | lm_SuperContig_0_v2:3893832-3895747 | 1017.78 | 9.83 | Catalase |
| Gene_6173 | Lema_T021470 | lm_SuperContig_1_v2:2566440-2566907 | 8702.49 | 9.61 | Hypothetical protein |
| Gene_9395 | Lema_T042140 | lm_SuperContig_4_v2:1136979-1138783 | 1287.62 | 9.58 | Alcohol dehydrogenase |
| Gene_11804 | Lema_T060560 | lm_SuperContig_8_v2:1317923-1319474 | 2035.40 | 9.20 | Hypothetical protein  SSP(Effector) |
| Gene_1799 | Lema_T087550 | lm_SuperContig_11_v2:702412-703074 | 1160.51 | 8.10 | Hypothetical protein  CSEP |
| Gene_11976 | Lema_T062280 | lm_SuperContig_8_v2:1760041-1760879 | 1715.93 | 7.82 | Fasciclin and related adhesion glycoproteins |
| Gene_6479 | Lema_T114240 | lm_SuperContig_20_v2:523704-524501 | 1909.61 | 7.52 | 3'-5'-exoribonuclease activity |
| Gene_3306 | Lema_T097730 | lm_SuperContig_14_v2:1208510-1208707 | 1669.20 | 7.31 | Hypothetical protein |
| Gene_5572 | Lema_T015460 | lm_SuperContig_1_v2:975571-976381 | 1421.12 | 7.00 | Hypothetical protein |
| Gene_2652 | Lema_T079530 | lm_SuperContig_13_v2:633177-634702 | 2647.31 | 6.94 | Hypothetical protein |
| Gene_7940 | Lema_T028340 | lm_SuperContig_2_v2:1614035-1614898 | 6817.24 | 6.67 | Transporter activity |
| Gene_616 | Lema_T006160 | lm_SuperContig_0_v2:2286680-2287371 | 1277.16 | 6.32 | Hypothetical protein |
| Gene_2276 | Lema_T085080 | lm_SuperContig_12_v2:978243-979186 | 7766.78 | 6.20 | Conserved Zn-finger protein |
| Gene_1516 | Lema_T075410 | lm_SuperContig_10_v2:1029046-1029213 | 26323.10 | 5.71 | Hypothetical protein |
| Gene_4933 | Lema_T109580 | lm_SuperContig_18_v2:974549-975828 | 3185.01 | 4.87 | Nucleic acid binding |
| Gene_6395 | Lema_T113400 | lm_SuperContig_20_v2:342998-344372 | 3091.05 | 4.79 | Nuclear pore complex |
| Gene_7977 | Lema_T028710 | lm_SuperContig_2_v2:1708344-1708944 | 4650.47 | 4.68 | Hypothetical protein |
| **7dpi** | | | | | |
| Gene_4884 | Lema_T109090 | lm_SuperContig_18_v2:862571-862990 | 6218.34 | 10.21 | Hypothetical protein |
| Gene_11624 | Lema_T058760 | lm_SuperContig_8_v2:828284-829346 | 745.96 | 9.23 | Hypothetical protein  SSP(Effector) |
| Gene_9395 | Lema_T042140 | lm_SuperContig_4_v2:1136979-1138783 | 737.81 | 8.67 | Alcohol dehydrogenase |
| Gene_1799 | Lema_T087550 | lm_SuperContig_11_v2:702412-703074 | 1079.07 | 7.89 | Hypothetical protein |
| Gene_7649 | Lema_T025430 | lm_SuperContig_2_v2:804099-805458 | 647.29 | 7.36 | Predicted transporter |
| Gene_6375 | Lema_T113200 | lm_SuperContig_20_v2:228262-228840 | 627.43 | 7.24 | Hypothetical protein  CSEP |
| Gene_6479 | Lema_T114240 | lm_SuperContig_20_v2:523704-524501 | 1437.33 | 7.01 | 3'-5'-exoribonuclease activity |
| Gene_11976 | Lema_T062280 | lm_SuperContig_8_v2:1760041-1760879 | 1047.93 | 7.01 | Fasciclin and related adhesion glycoproteins |
| Gene_6173 | Lema_T021470 | lm_SuperContig_1_v2:2566440-2566907 | 1451.01 | 6.92 | Hypothetical protein |
| Gene_616 | Lema_T006160 | lm_SuperContig_0_v2:2286680-2287371 | 1077.12 | 5.97 | Hypothetical protein |
| Gene_3811 | Lema_T093240 | lm_SuperContig_15_v2:1293850-1295649 | 523.07 | 5.96 | Transporter activity |
| Gene_1613 | Lema_T076380 | lm_SuperContig_10_v2:1509441-1509941 | 1667.90 | 5.41 | Hypothetical protein  CSEP |
| Gene_2652 | Lema_T079530 | lm_SuperContig_13_v2:633177-634702 | 885.44 | 5.26 | Hypothetical protein |
| Gene_5266 | Lema_T112910 | lm_SuperContig_19_v2:920520-920711 | 627.36 | 5.25 | Hypothetical protein |
| Gene_6138 | Lema_T021120 | lm_SuperContig_1_v2:2476727-2477421 | 514.33 | 5.18 | Hypothetical protein |
| Gene_234 | Lema_T002340 | lm_SuperContig_0_v2:882882-883215 | 1472.66 | 5.01 | Hypothetical protein |
| Gene_6395 | Lema_T113400 | lm_SuperContig_20_v2:342998-344372 | 3526.32 | 4.88 | Nuclear pore complex |
| Gene_317 | Lema_T003170 | lm_SuperContig_0_v2:1132285-1135705 | 675.60 | 4.49 | Hypothetical protein |
| Gene_6046 | Lema_T020200 | lm_SuperContig_1_v2:2288535-2288783 | 950.68 | 4.01 | Hypothetical protein |
| Gene_10764 | Lema_T049500 | lm_SuperContig_6_v2:1208619-1209139 | 861.15 | 3.73 | Hypothetical protein |
| **11dpi** | | | | | |
| Gene_4884 | Lema_T109090 | lm_SuperContig_18_v2:862571-862990 | 2754.46 | 10.30 | Hypothetical protein |
| Gene_3471 | Lema_T089840 | lm_SuperContig_15_v2:160448-161311 | 572.41 | 8.91 | Cutinase/catalytic activity |
| Gene_1799 | Lema_T087550 | lm_SuperContig_11_v2:702412-703074 | 697.08 | 8.53 | Hypothetical protein  CSEP |
| Gene_11976 | Lema_T062280 | lm_SuperContig_8_v2:1760041-1760879 | 704.79 | 7.71 | Fasciclin and related adhesion glycoproteins (KOG1437) |
| Gene_6173 | Lema_T021470 | lm_SuperContig_1_v2:2566440-2566907 | 987.23 | 7.63 | Hypothetical protein |
| Gene_616 | Lema_T006160 | lm_SuperContig_0_v2:2286680-2287371 | 1315.65 | 7.52 | Hypothetical protein |
| Gene_6138 | Lema_T021120 | lm_SuperContig_1_v2:2476727-2477421 | 543.49 | 6.52 | Hypothetical protein |
| Gene_317 | Lema_T003170 | lm_SuperContig_0_v2:1132285-1135705 | 661.81 | 5.74 | Hypothetical protein |
| Gene_7608 | Lema_T025020 | lm_SuperContig_2_v2:573218-575068 | 713.76 | 5.59 | Splicing coactivator |
| Gene_9092 | Lema_T039110 | lm_SuperContig_4_v2:302019-302430 | 1072.50 | 5.42 | Heat shock protein  response to stress |
| Gene_1516 | Lema_T075410 | lm_SuperContig_10_v2:1029046-1029213 | 6393.46 | 4.83 | Hypothetical protein |
| Gene_1613 | Lema_T076380 | lm_SuperContig_10_v2:1509441-1509941 | 435.35 | 4.71 | Hypothetical protein  CSEP |
| Gene_9685 | Lema_T050140 | lm_SuperContig_5_v2:77779-80386 | 504.20 | 3.91 | WD40 repeat protein |
| Gene_6395 | Lema_T113400 | lm_SuperContig_20_v2:342998-344372 | 737.42 | 3.89 | Nuclear pore complex |
| Gene_5078 | Lema_T111030 | lm_SuperContig_19_v2:321482-322624 | 583.54 | 3.29 | Carbon-sulfur lyase activity |
| Gene_6925 | Lema_T118700 | lm_SuperContig_21_v2:939831-940340 | 560.09 | 3.15 | Hypothetical protein |
| Gene_7728 | Lema_T026220 | lm_SuperContig_2_v2:961066-962667 | 673.63 | 2.61 | Hypothetical protein |
| Gene_7977 | Lema_T028710 | lm_SuperContig_2_v2:1708344-1708944 | 420.46 | 2.38 | Hypothetical protein |
| Gene_10737 | Lema_T049230 | lm_SuperContig_6_v2:1147075-1147714 | 1554.12 | 2.33 | Ubiquinol cytochrome c reductase |
| Gene_9900 | Lema_T052290 | lm_SuperContig_5_v2:796981-797687 | 597.75 | 2.15 | Serine/threonine protein phosphatase 2A |
| Gene_3726 | Lema_T092390 | lm_SuperContig_15_v2:1048818-1049323 | 711.46 | 2.12 | Protein of unknown function DUF1960 |
| Gene_8224 | Lema_T031180 | lm_SuperContig_2_v2:2330119-2330586 | 633.78 | 2.03 | Putative translation initiation inhibitor |
