# Supplementary material for: Comparative Transcriptomic Analysis of Virulence Factors in Leptosphaeria maculans during Compatible and Incompatible Interactions with Canola
Source: Front Plant Sci. 2016 Dec 1;7:1784. doi: 10.3389/fpls.2016.01784 (PMC5131014; doi:10.3389/fpls.2016.01784)
Supplement: Supplementary file 6 [file Table6.DOCX]

**Supplementary Table 6**. Upregulated genes representing six classes of CAZymes observed at different developmental stages of *Leptosphaeria maculans* during compatible and incompatible interactions

| CAZyme families | Topas-wild | | | | Topas-*Rlm2* | | | |
| --- | --- | --- | --- | --- | --- | --- | --- | --- |
|  | 3 dpi | 5 dpi | 7 dpi | 11 dpi | 3 dpi | 5 dpi | 7 dpi | 11 dpi |
| Glycoside Hydrolase(GH) | 35 | 13 | 66 | 103 | 39 | 29 | 28 | 45 |
| Carbohydrate Esterase(CE) | 10 | 5 | 17 | 44 | 12 | 7 | 6 | 12 |
| Auxiliary Activities(AA) | 13 | 2 | 15 | 31 | 13 | 6 | 9 | 12 |
| GlycosylTransferase(GT) | 18 | 0 | 20 | 26 | 22 | 6 | 8 | 3 |
| Polysaccharide Lyase (PL) | 6 | 1 | 9 | 7 | 7 | 3 | 7 | 6 |
| Carbohydrate-Binding Module(CBM) | 4 | 2 | 7 | 15 | 8 | 5 | 5 | 4 |
