# Supplementary material for: Comparative Transcriptomic Analysis of Virulence Factors in Leptosphaeria maculans during Compatible and Incompatible Interactions with Canola
Source: Front Plant Sci. 2016 Dec 1;7:1784. doi: 10.3389/fpls.2016.01784 (PMC5131014; doi:10.3389/fpls.2016.01784)
Supplement: Supplementary file 9 [file Image1.PDF]

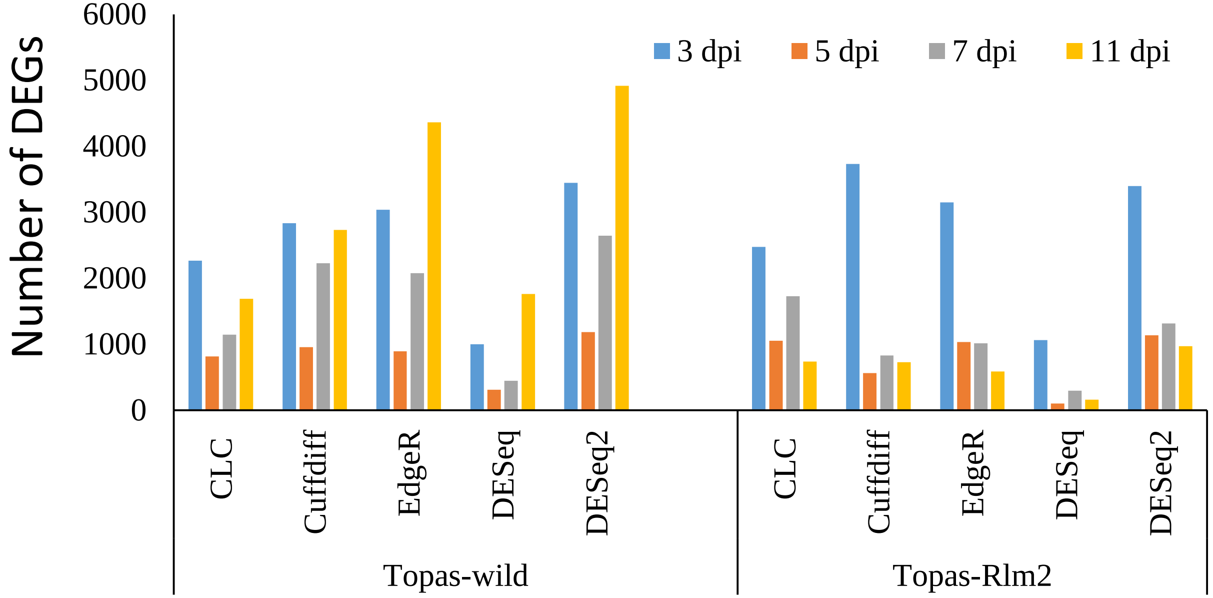

Supplementary Figure 1. Number of differentially expressed genes (DEGs) identified using different software tools at four inplanta developmental stages of *Leptosphaeria maculans* compared to axenic culture.
