# Supplementary material for: Comparative Transcriptomic Analysis of Virulence Factors in Leptosphaeria maculans during Compatible and Incompatible Interactions with Canola
Source: Front Plant Sci. 2016 Dec 1;7:1784. doi: 10.3389/fpls.2016.01784 (PMC5131014; doi:10.3389/fpls.2016.01784)
Supplement: Supplementary file 10 [file Image2.PDF]

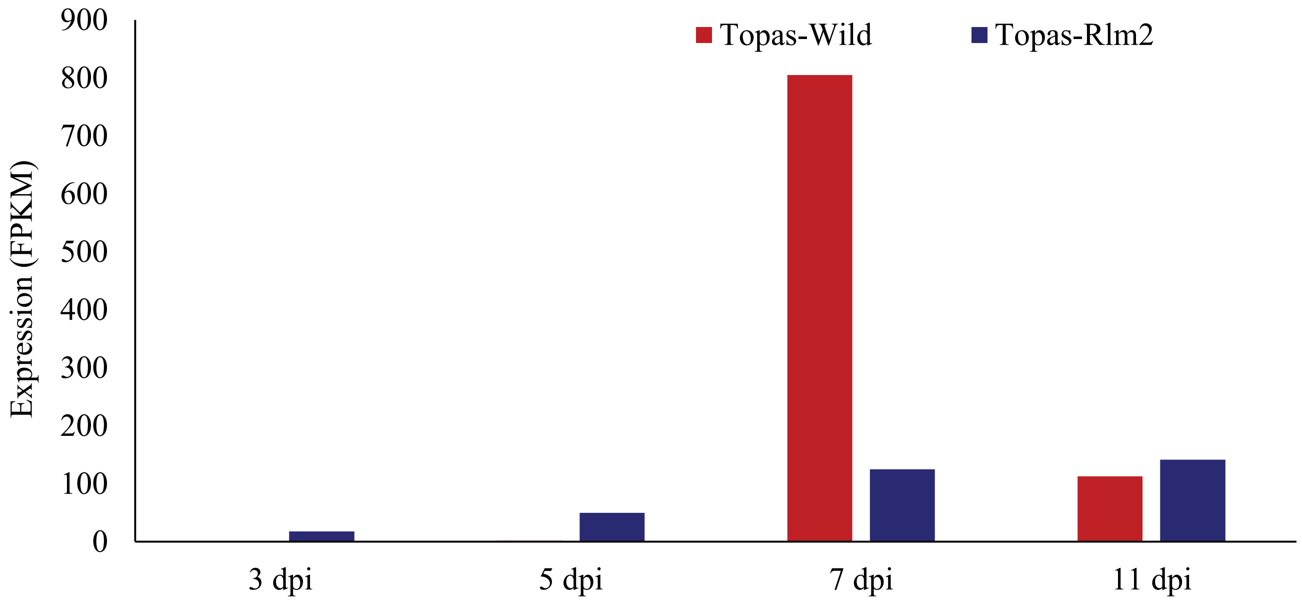

Supplementary Figure 2. Expression pattern of known Crinkler gene at different disease developmental stages in Leptosphaeria maculans during compatible (Topas-wild) and incompatible (Topas-Rlm2) interactions. FPKM- Fragments per kilo-base of transcript per million mapped reads; dpi - days post inoculation. Analyses were performed with five biological replicates.
