# Supplementary material for: Comparative Transcriptomic Analysis of Virulence Factors in Leptosphaeria maculans during Compatible and Incompatible Interactions with Canola
Source: Front Plant Sci. 2016 Dec 1;7:1784. doi: 10.3389/fpls.2016.01784 (PMC5131014; doi:10.3389/fpls.2016.01784)
Supplement: Supplementary file 11 [file Image3.PDF]

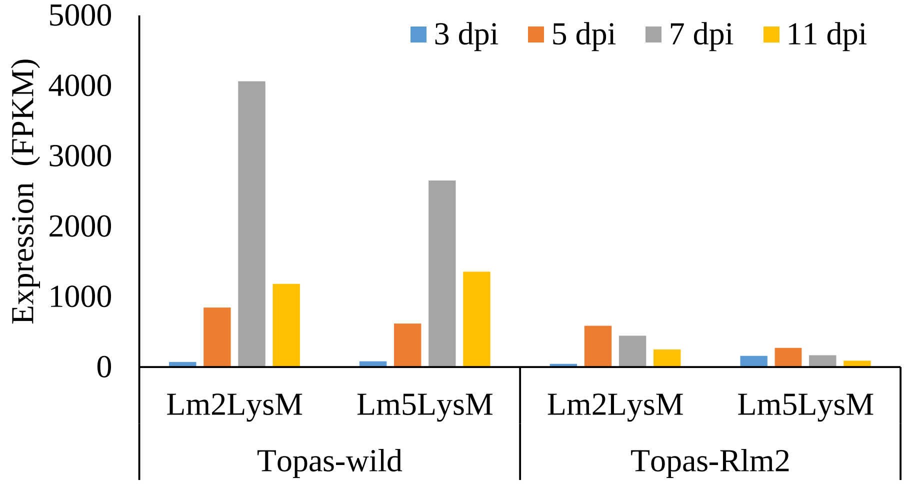

Supplementary Figure 3. Expression pattern of *LysM* genes at different disease developmental stages in *Leptosphaeria maculans* during compatible and incompatible interactions**.** FPKM- Fragments per kilo-base of transcript per million mapped reads; dpi - days post inoculation. Analyses were performed with five biological replicates
