# Supplementary material for: Comparative Transcriptomic Analysis of Virulence Factors in Leptosphaeria maculans during Compatible and Incompatible Interactions with Canola
Source: Front Plant Sci. 2016 Dec 1;7:1784. doi: 10.3389/fpls.2016.01784 (PMC5131014; doi:10.3389/fpls.2016.01784)
Supplement: Supplementary file 12 [file Image4.PDF]

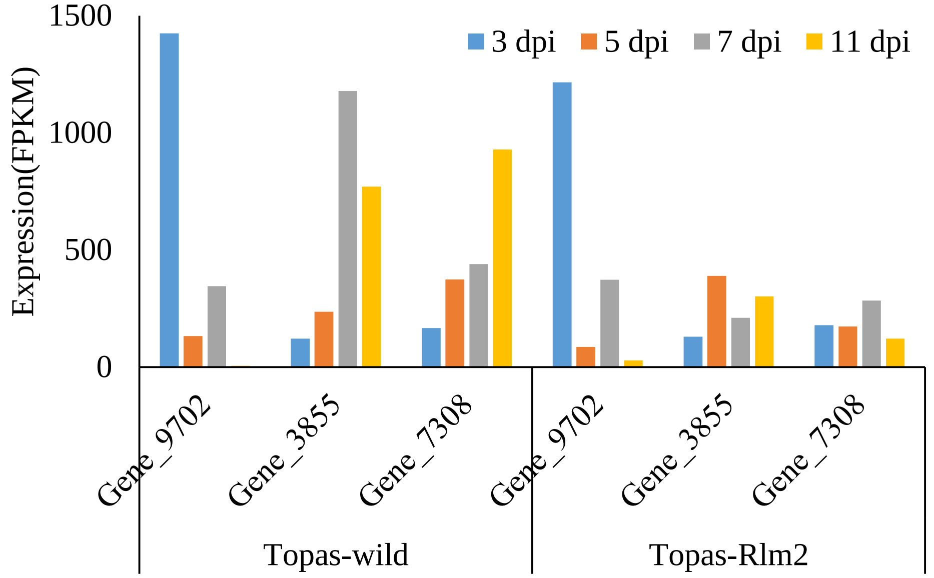

Supplementary Figure 4. Expression pattern of representative CAZymes at early, mid and late growth stages of *Leptosphaeria maculans* during interactions with canola compatible host Topas-wild and incompatible host Topas-*Rlm2*. Analyses were performed with five biological replicates. dpi- days post inoculations, FPKM- Fragments per kilo-base of transcript per million mapped reads
