# Supplementary material for: Comparative Transcriptomic Analysis of Virulence Factors in Leptosphaeria maculans during Compatible and Incompatible Interactions with Canola
Source: Front Plant Sci. 2016 Dec 1;7:1784. doi: 10.3389/fpls.2016.01784 (PMC5131014; doi:10.3389/fpls.2016.01784)
Supplement: Supplementary file 13 [file Image5.PDF]

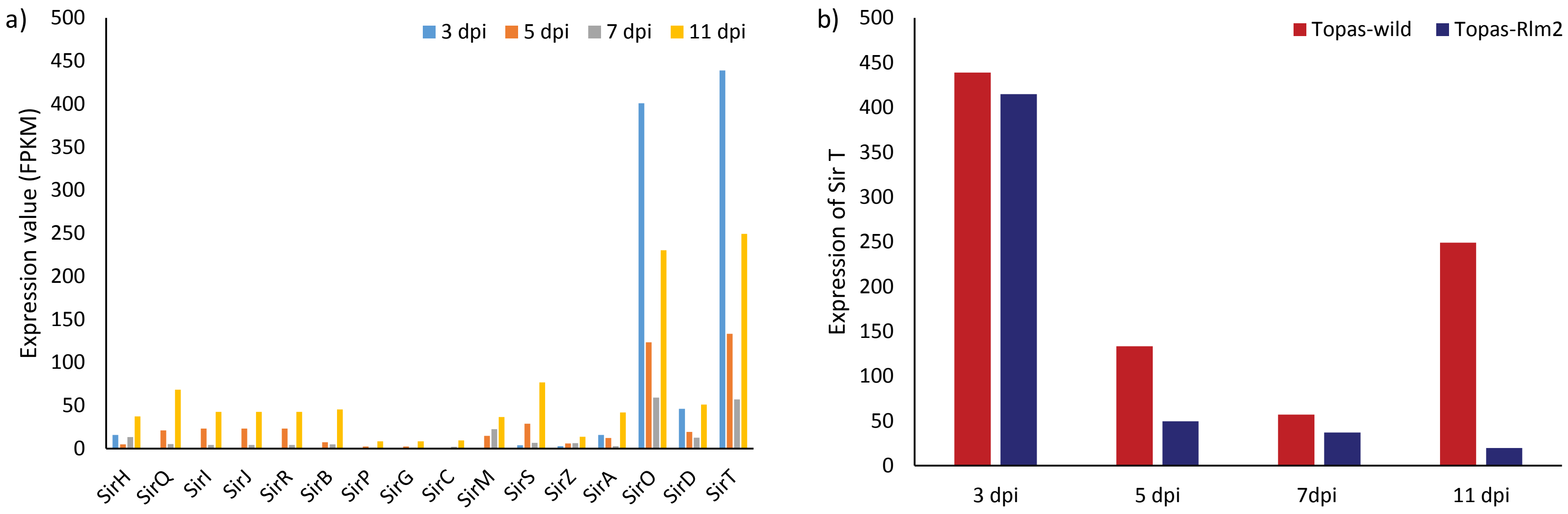

Supplementary Figure 5. (a) Expression of 16 *Leptosphaeria maculans sirodesmin* genes at 3, 5, 7 and 11 dpi during compatible interaction of *L. maculans*. (b) Expression pattern of SirT gene during compatible (Topas-wild) and incompatible interaction (Topas-Rlm2). dpi- days post inoculations, FPKM- Fragments per kilo-base of transcript per million mapped reads. Analyses were performed with five biological replicates.
