# Supplementary material for: Comparative Transcriptomic Analysis of Virulence Factors in Leptosphaeria maculans during Compatible and Incompatible Interactions with Canola
Source: Front Plant Sci. 2016 Dec 1;7:1784. doi: 10.3389/fpls.2016.01784 (PMC5131014; doi:10.3389/fpls.2016.01784)
Supplement: Supplementary file 14 [file Image6.PDF]

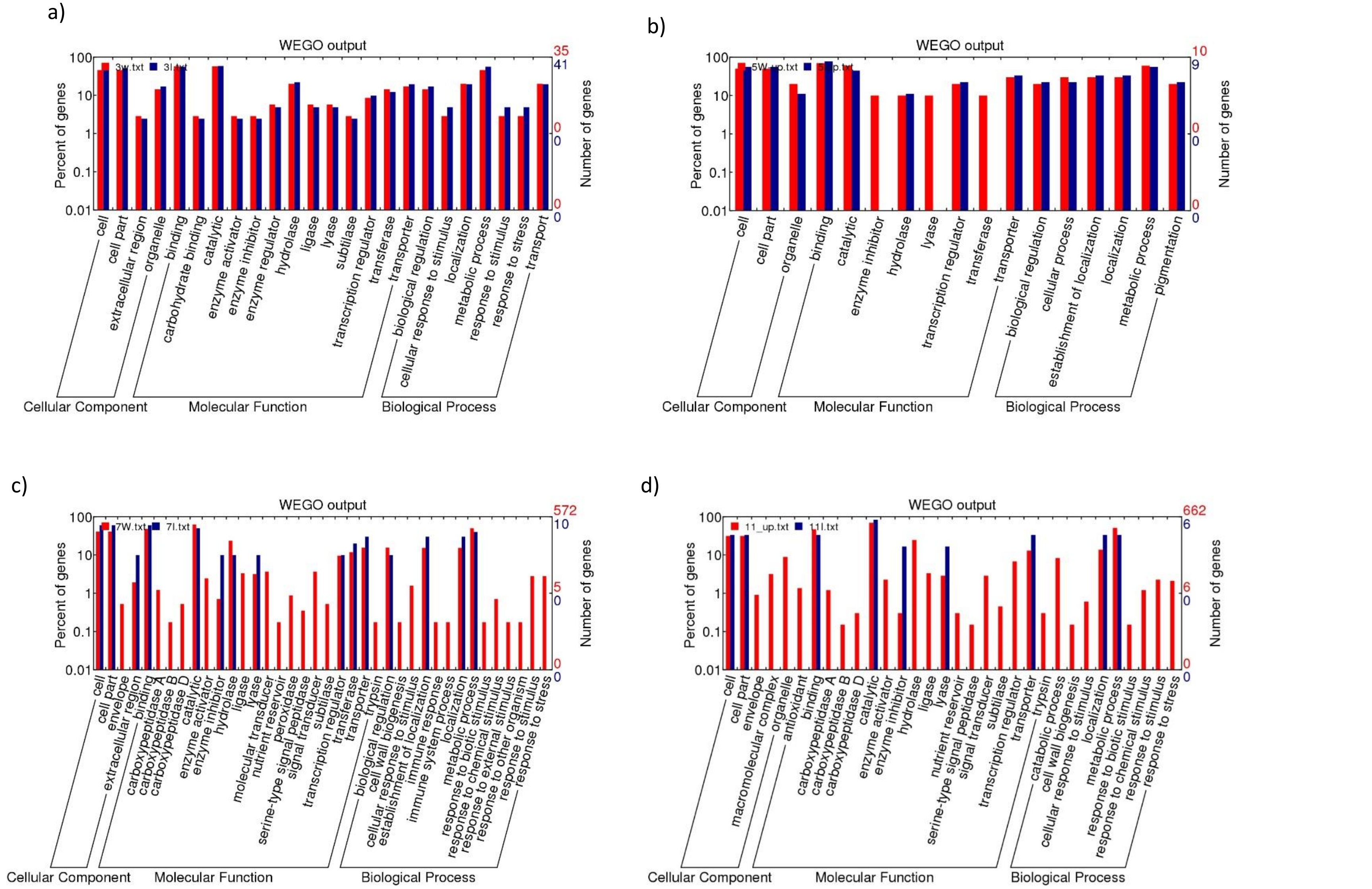

Supplementary Figure 6. Functional categorization of up-regulated genes at a) 3 dpi, b) 5 dpi, c) 7 dpi, and d) 11 dpi during *Leptosphaeria maculans* compatible (red) and incompatible (blue) interactions. The right y-axis indicates the number of genes in a

category. The left y-axis indicates the percentage of a specific category of genes in the main category. Functional categorization was performed based on gene ontology term assigned by WEGO software tool.
