# Supplementary material for: Comparative Transcriptomic Analysis of Virulence Factors in Leptosphaeria maculans during Compatible and Incompatible Interactions with Canola
Source: Front Plant Sci. 2016 Dec 1;7:1784. doi: 10.3389/fpls.2016.01784 (PMC5131014; doi:10.3389/fpls.2016.01784)
Supplement: Supplementary file 15 [file Image7.PDF]

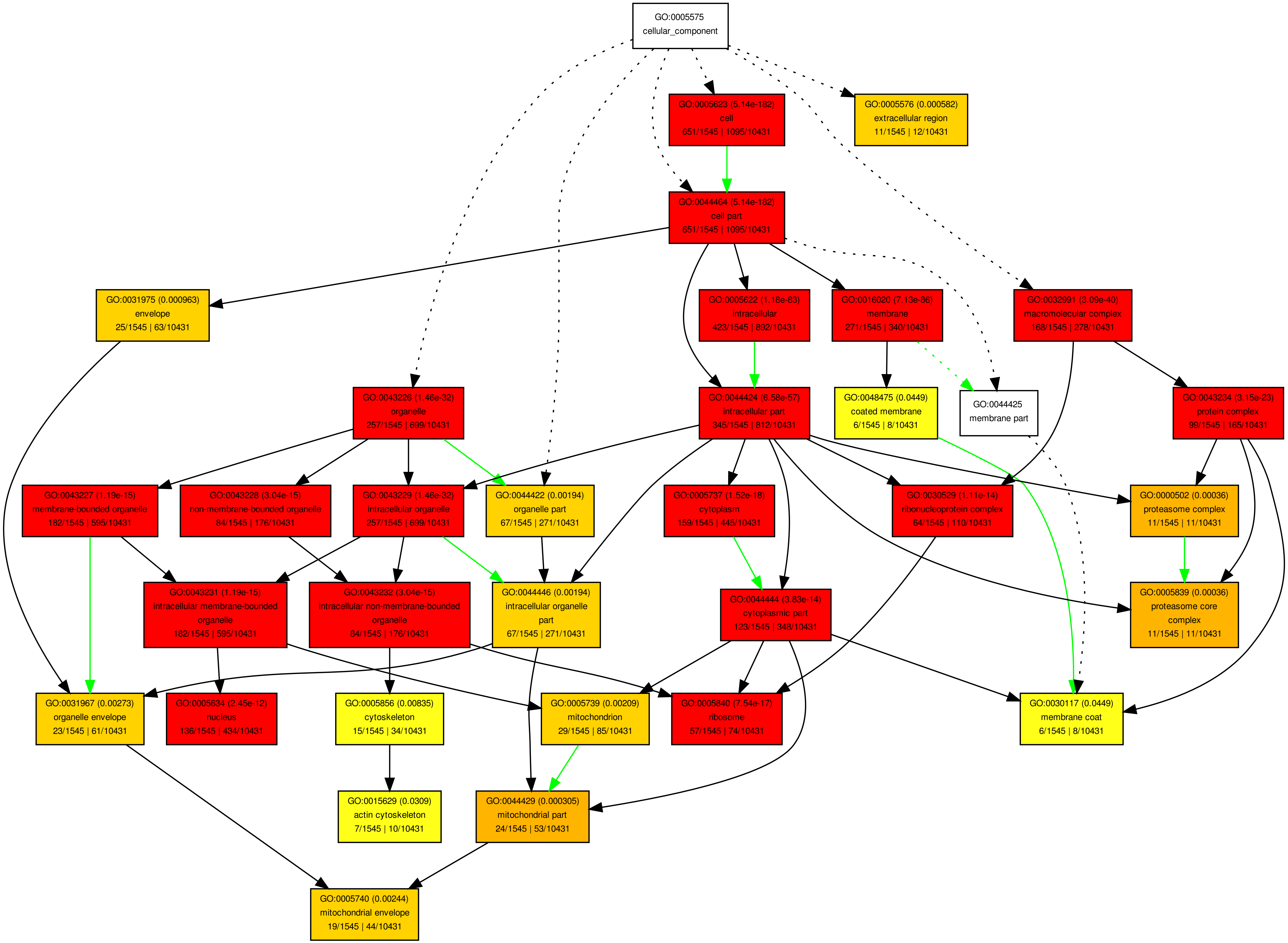
GO:0005575
cellular_component
GO:0005623 (5.14e-182)
cell
651/1545 | 1095/10431
GO:0005576 (0.000582)
extracellular region
11/1545 | 12/10431
GO:0044464 (5.14e-182)
cell part
651/1545 | 1095/10431
GO:0031975 (0.000963)
envelope
25/1545 | 63/10431
GO:0005622 (1.18e-83)
intracellular
423/1545 | 892/10431
GO:0016020 (7.13e-86)
membrane
271/1545 | 340/10431
GO:0032991 (3.09e-40)
macromolecular complex
168/1545 | 278/10431
GO:0043226 (1.46e-32)
organelle
257/1545 | 699/10431
GO:0044424 (6.58e-57)
intracellular part
345/1545 | 812/10431
GO:0048475 (0.0449)
coated membrane
6/1545 | 8/10431
GO:0044425
membrane part
GO:0043234 (3.15e-23)
protein complex
99/1545 | 165/10431
GO:0043227 (1.19e-15)
membrane-bounded organelle
182/1545 | 595/10431
GO:0043228 (3.04e-15)
non-membrane-bounded organelle
84/1545 | 176/10431
GO:0043229 (1.46e-32)
intracellular organelle
257/1545 | 699/10431
GO:0044422 (0.00194)
organelle part
67/1545 | 271/10431
GO:0005737 (1.52e-18)
cytoplasm
159/1545 | 445/10431
GO:0030529 (1.11e-14)
ribonucleoprotein complex
64/1545 | 110/10431
GO:0000502 (0.00036)
proteasome complex
11/1545 | 11/10431
GO:0043231 (1.19e-15)
intracellular membrane-bounded
organelle
182/1545 | 595/10431
GO:0043232 (3.04e-15)
intracellular non-membrane-bounded
organelle
84/1545 | 176/10431
GO:0044446 (0.00194)
intracellular organelle
part
67/1545 | 271/10431
GO:0044444 (3.83e-14)
cytoplasmic part
123/1545 | 348/10431
GO:0005839 (0.00036)
proteasome core
complex
11/1545 | 11/10431
GO:0031967 (0.00273)
organelle envelope
23/1545 | 61/10431
GO:0005634 (2.45e-12)
nucleus
136/1545 | 434/10431
GO:0005856 (0.00835)
cytoskeleton
15/1545 | 34/10431
GO:0005739 (0.00209)
mitochondrion
29/1545 | 85/10431
GO:0005840 (7.54e-17)
ribosome
57/1545 | 74/10431
GO:0030117 (0.0449)
membrane coat
6/1545 | 8/10431
GO:0015629 (0.0309)
actin cytoskeleton
7/1545 | 10/10431
GO:0044429 (0.000305)
mitochondrial part
24/1545 | 53/10431
GO:0005740 (0.00244)
mitochondrial envelope
19/1545 | 44/10431

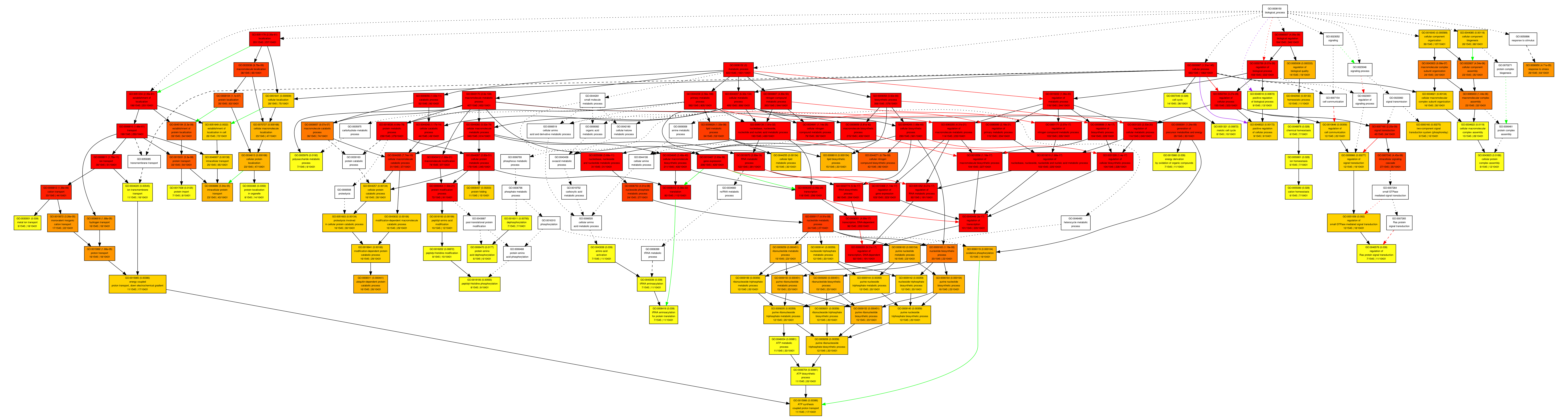

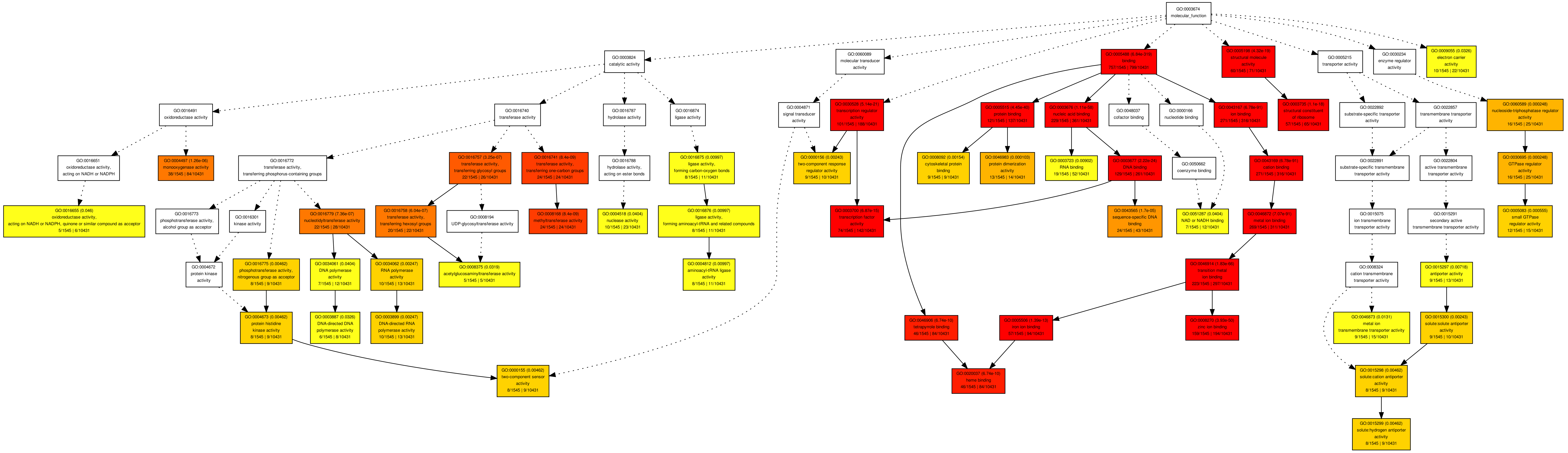
GO:0003674 molecular_function
GO:0003824 catalytic activity
GO:0060089 molecular transducer activity
GO:0005488 (6.84e-319) binding 757/1545 | 799/10431
GO:0005198 (4.32e-19) structural molecule activity 60/1545 | 71/10431
GO:0005215 transporter activity
GO:0030234 enzyme regulator activity
GO:0009055 (0.0326) electron carrier activity 10/1545 | 22/10431
GO:0016491 oxidoreductase activity
GO:0016740 transferase activity
GO:0016787 hydrolase activity
GO:0016874 ligase activity
GO:0004871 signal transducer activity
GO:0030528 (5.14e-21) transcription regulator activity 101/1545 | 188/10431
GO:0005515 (4.45e-40) protein binding 121/1545 | 137/10431
GO:0003676 (1.11e-58) nucleic acid binding 229/1545 | 361/10431
GO:0048037 cofactor binding
GO:0000166 nucleotide binding
GO:0043167 (6.78e-91) ion binding 271/1545 | 316/10431
GO:0003735 (1.1e-18) structural constituent of ribosome 57/1545 | 65/10431
GO:0022892 substrate-specific transporter activity
GO:0022857 transmembrane transporter activity
GO:0060589 (0.000248) nucleoside-triphosphatase regulator activity 16/1545 | 25/10431
GO:0016651 oxidoreductase activity, acting on NADH or NADPH
GO:0004497 (1.26e-06) monooxygenase activity 38/1545 | 84/10431
GO:0016772 transferase activity, transferring phosphorus-containing groups
GO:0016757 (3.25e-07) transferase activity, transferring glycosyl groups 22/1545 | 26/10431
GO:0016741 (8.4e-09) transferase activity, transferring one-carbon groups 24/1545 | 24/10431
GO:0016788 hydrolase activity, acting on ester bonds
GO:0016875 (0.00997) ligase activity, forming carbon-oxygen bonds 8/1545 | 11/10431
GO:0000156 (0.00243) two-component response regulator activity 9/1545 | 10/10431
GO:0008092 (0.00154) cytoskeletal protein binding 9/1545 | 9/10431
GO:0046983 (0.000103) protein dimerization activity 13/1545 | 14/10431
GO:0003723 (0.00902) RNA binding 19/1545 | 52/10431
GO:0003677 (2.22e-24) DNA binding 129/1545 | 261/10431
GO:0050662 coenzyme binding
GO:0043169 (6.78e-91) cation binding 271/1545 | 316/10431
GO:0022891 substrate-specific transmembrane transporter activity
GO:0022804 active transmembrane transporter activity
GO:0030695 (0.000248) GTPase regulator activity 16/1545 | 25/10431
GO:0016655 (0.046) oxidoreductase activity, acting on NADH or NADPH, quinone or similar compound as acceptor 5/1545 | 6/10431
GO:0016773 phosphotransferase activity, alcohol group as acceptor
GO:0016301 kinase activity
GO:0016779 (7.36e-07) nucleotidyltransferase activity 22/1545 | 28/10431
GO:0016758 (6.04e-07) transferase activity, transferring hexosyl groups 20/1545 | 22/10431
GO:0008194 UDP-glycosyltransferase activity
GO:0008168 (8.4e-09) methyltransferase activity 24/1545 | 24/10431
GO:0004518 (0.0404) nuclease activity 10/1545 | 23/10431
GO:0016876 (0.00997) ligase activity, forming aminoacyl-tRNA and related compounds 8/1545 | 11/10431
GO:0003700 (6.87e-15) transcription factor activity 74/1545 | 142/10431
GO:0043565 (1.7e-05) sequence-specific DNA binding 24/1545 | 43/10431
GO:0051287 (0.0404) NAD or NADH binding 7/1545 | 12/10431
GO:0046872 (7.07e-91) metal ion binding 269/1545 | 311/10431
GO:0015075 ion transmembrane transporter activity
GO:0015291 secondary active transmembrane transporter activity
GO:0005083 (0.000555) small GTPase regulator activity 12/1545 | 15/10431
GO:0004672 protein kinase activity
GO:0016775 (0.00462) phosphotransferase activity, nitrogenous group as acceptor 8/1545 | 9/10431
GO:0034061 (0.0404) DNA polymerase activity 7/1545 | 12/10431
GO:0034062 (0.00247) RNA polymerase activity 10/1545 | 13/10431
GO:0008375 (0.0319) acetylglucosaminyltransferase activity 5/1545 | 5/10431
GO:0004812 (0.00997) aminoacyl-tRNA ligase activity 8/1545 | 11/10431
GO:0046914 (1.83e-66) transition metal ion binding 223/1545 | 297/10431
GO:0008324 cation transmembrane transporter activity
GO:0015297 (0.00718) antiporter activity 9/1545 | 13/10431
GO:0004673 (0.00462) protein histidine kinase activity 8/1545 | 9/10431
GO:0003887 (0.0326) DNA-directed DNA polymerase activity 6/1545 | 8/10431
GO:0003899 (0.00247) DNA-directed RNA polymerase activity 10/1545 | 13/10431
GO:0046906 (6.74e-10) tetrapyrrole binding 46/1545 | 84/10431
GO:0005506 (1.39e-13) iron ion binding 57/1545 | 94/10431
GO:0008270 (3.93e-50) zinc ion binding 159/1545 | 194/10431
GO:0046873 (0.0131) metal ion transmembrane transporter activity 9/1545 | 15/10431
GO:0015300 (0.00243) solute:solute antiporter activity 9/1545 | 10/10431
GO:0000155 (0.00462) two-component sensor activity 8/1545 | 9/10431
GO:0020037 (6.74e-10) heme binding 46/1545 | 84/10431
GO:0015298 (0.00462) solute:cation antiporter activity 8/1545 | 9/10431
GO:0015299 (0.00462) solute:hydrogen antiporter activity 8/1545 | 9/10431

Supplementary Figure 7. AgriGO results of genes differentially expressed during Leptosphaeria maculans compatible interactions at 7 dpi. The DEGs were grouped into three main classes a) cellular components b) biological process and c) molecular functions. Significance level of enrichment is displayed by color scale, where white indicates no significant enrichment, yellow and red indicates strength of significance.
